# WNT Signaling in Melanoma

**DOI:** 10.3390/ijms21144852

**Published:** 2020-07-09

**Authors:** Anna Gajos-Michniewicz, Malgorzata Czyz

**Affiliations:** Department of Molecular Biology of Cancer, Medical University of Lodz, 6/8 Mazowiecka Street, 92–215 Lodz, Poland; anna.gajos-michniewicz@umed.lodz.pl

**Keywords:** WNT, β-catenin, WNT5A, melanoma, immune evasion, signal transduction crosstalk

## Abstract

WNT-signaling controls important cellular processes throughout embryonic development and adult life, so any deregulation of this signaling can result in a wide range of pathologies, including cancer. WNT-signaling is classified into two categories: β-catenin-dependent signaling (canonical pathway) and β-catenin-independent signaling (non-canonical pathway), the latter can be further divided into WNT/planar cell polarity (PCP) and calcium pathways. WNT ligands are considered as unique directional growth factors that contribute to both cell proliferation and polarity. Origin of cancer can be diverse and therefore tissue-specific differences can be found in WNT-signaling between cancers, including specific mutations contributing to cancer development. This review focuses on the role of the WNT-signaling pathway in melanoma. The current view on the role of WNT-signaling in cancer immunity as well as a short summary of WNT pathway-related drugs under investigation are also provided.

## 1. Introduction

The study of WNT-signaling was initiated in the early 1980′s by the discovery of *Wingless*, a *Drosophila* segment polarity gene [1] and then the mouse proto-oncogene *Int1* [2]. The term ‘WNT’ comes from a combination of these two names of the same gene [3]. The WNT-signaling is evolutionarily conserved and plays an important role in the embryonic development, adult tissue homeostasis and regeneration [4]. Furthermore, it maintains genetic stability and is important for cell fate and differentiation, cell proliferation, cell motility, apoptosis and stem cell maintenance [5]. Aberrant functioning of WNT-signaling is associated with a number of diseases, including embryonic malformations, degenerative diseases and cancer [6,7,8,9]. WNT-signaling is divided into two pathways: β-catenin-dependent also known as canonical or WNT/β-catenin pathway and β-catenin-independent—also termed as non-canonical—which can be further divided into WNT/planar cell polarity (PCP) and calcium pathway that in some circumstances can antagonize WNT/β-catenin-signaling [10]. The β-catenin-dependent pathway mainly controls cell proliferation, whereas β-catenin-independent signaling regulates cell polarity and migration. This distinction, however, is conventional as these two main pathways form a network with concomitant crosstalk and mutual regulation [11,12]. Better understanding of the mechanisms that govern the highly context-dependent outcome of WNT-signaling in different tumors is important for the development of appropriate treatment strategies. This review is focused on WNT-signaling in melanoma, a tumor derived from melanocytes that arise from neural crest cells.

### 1.1. WNT Ligands in Canonical and Non-Canonical WNT Signaling Pathways

**Figure 1:**
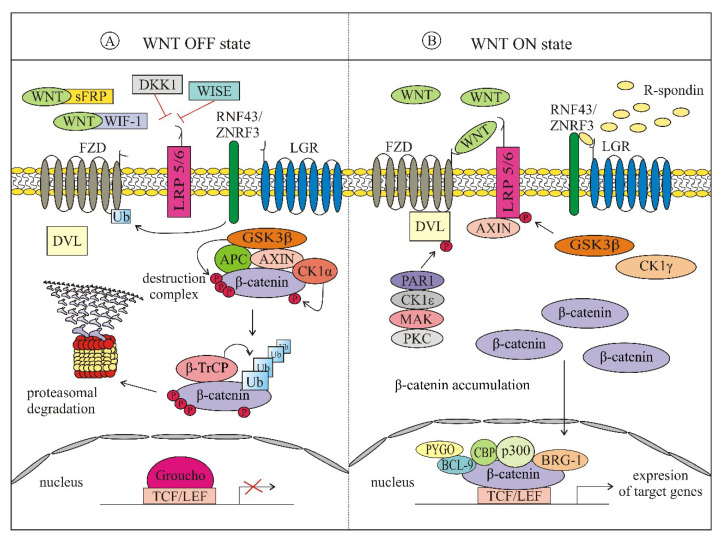
Simplified scheme of canonical WNT -signaling pathway. (**A**) In the absence of WNT ligands (WNT OFF state), β-catenin is phosphorylated by a destruction complex consisting of AXIN, APC, GSK3β and CK1α to be further ubiquitinated for proteasomal degradation. In the absence of R-spondins, E3 ubiquitin ligases RNF43/ZNRF3 target FZD for lysosomal degradation; (**B**) binding of WNT ligands to FZD receptors and LRP co-receptors activates WNT-signaling (WNT ON state). AXIN is associated with LRP5/6, whereas DVL is recruited to FZD, which results in dissociation of the destructive complex. β-catenin is accumulated and stabilized in the cytosol, and then unphosphorylated β-catenin is translocated to the nucleus to activate the expression of WNT target genes. APC—adenomatosis polyposis coli; AXIN—axis inhibition protein; BCL—B-cell CLL/lymphoma protein; BRG-1—brahma-related gene-1; CBP—(CREB)-binding protein; CK1α—casein kinase 1α; CK1γ—casein kinase 1γ; CK1ε—casein kinase 1ε; DKK1—Dickkopf-1; DVL—disheveled; FZD—frizzled receptor; GSK3β—glycogen synthase kinase 3β; LEF—lymphoid enhancer-binding factor 1; LGR—leucine-rich repeat-containing G-protein coupled receptor; LRP—low-density lipoprotein receptor related protein; MAK—metastasis associated kinase; PAR1—protease-activated receptor 1; PKC—protein kinase C; PYGO—pygopus; RNF43—ring finger protein 43; sFRP—secreted frizzled-related proteins; TCF—T cell factor; β-TrCP—beta-transducin repeats-containing proteins; WIF1—WNT inhibitory factor 1; WISE—WNT modulator in surface ectoderm; Ub; ubiquitin; ZNRF3—zinc and ring finger protein 3.

**Figure 2:**
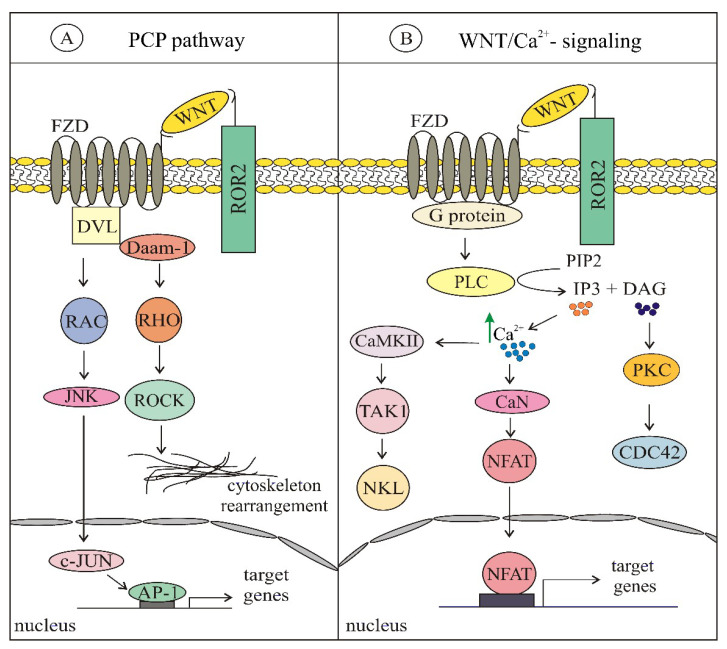
An overview of non-canonical WNT-signaling pathways: (**A**) WNT/planar cell polarity-signaling pathway (PCP) is initiated by WNT binding to FZD and ROR, then DVL is recruited and DVL-Daam-1 complex is activated, followed by JNK and ROCK activation and cytoskeletal rearrangement; (**B**) WNT/Ca^2+^-signaling pathway is initiated by WNT binding to FZD and ROR, with further G-protein triggered phospholipase C activation leading to phospholipase C intracellular calcium fluxes and downstream calcium dependent responses. AP-1—activator protein 1; CaMKII—Ca2+/calmodulin dependent kinase II; CaN—calcineurin; CDC42—cell division cycle 42; DAG—diacylglycerol; DAAM1—DVL associated activator of morphogenesis; DVL—disheveled; FZD—frizzled; JNK—c-Jun N-terminal kinases; NLK—nemo like kinase; NFAT—nuclear factor of activated T-cells; PIP2—phosphatidylinositol (4,5)-biphosphates; PKC—protein kinase C; PLC—phospholipase C; RAC—Ras-related C3 botulinum toxin substrate; RHO—Ras homolog gene family; ROCK—Rho-associated kinase; ROR—RAR-related orphan receptor; TAK1—transforming growth factor beta-activated kinase 1.

The WNT family of secreted proteins includes 19 cysteine-rich glycoproteins (~40 kDa; ~350–400 amino acids with a 20–85% sequence identity) [4,13], in which postranslational modifications comprising glycosylation and palmitoylation are considered to be essential for their biologic activity [6,14]. Porcupine, endoplasmic reticulum resident acyltransferase, is the enzyme that is required for the attachment of palmitoleic acid to WNT ligands [6,8,14]. Then, WNT ligands bind to an evolutionary highly conserved transmembrane protein Evenness interrupted/Wntless (EVI/WLS) and are shuttled to the plasma membrane via the Golgi apparatus [15]. By clathrin-mediated endocytosis, EVI/WLS is recycled in the Golgi apparatus by the retromer complex. There are several routes enabling WNT proteins to exit the cells: by solubilization, exosome formation or by lipoprotein particles (LPPs), serving as extracellular transporters to achieve long-range signaling [4,8,15]. The interactions between WNTs and their specific receptors activate WNT pathways: canonical (β-catenin-dependent) (Figure 1) and non-canonical (β-catenin-independent) (Figure 2) that cooperate with each other in regulation of important cellular processes. Generally, the ligand subtype determines the mode of the WNT-signaling network. WNT1, WNT2, WNT3, WNT3A, WNT8a, WNT8b, WNT10a and WNT10b are activators of the canonical pathway, whereas WNT4, WNT5A, WNT5B, WNT6, WNT7a, WNT7b and WNT11 are common activators of non-canonical WNT-signaling [16,17]. WNTs are classified as directional growth factors with unique properties since they influence proliferation and polarity, and both may occur at the same time and in the same cells [18]. Moreover, WNTs can act in an autocrine and paracrine manner [6,19,20].

### 1.2. β-Catenin-Dependent (Canonical) WNT Signaling

In resting cells, in the absence of activating signals (Figure 1A), the level of β-catenin is low, which is achieved by the cytoplasmic ‘destruction complex’ that consists of axis inhibition protein 1 (AXIN1), adenomatosis polyposis coli (APC), glycogen synthase kinase 3β (GSK3β) and casein kinase 1α (CK1α) [7,21,22,23]. AXIN1 is the central scaffold protein, which directly interacts with all other core components of the destruction complex [24]. It is a concentration-limiting protein [25], and its cellular level is stabilized by SUMOylation [26] and decreased by degradation involving activated low-density lipoprotein receptor related protein 5/6 (LRP5/6) [27] and tankyrases [28]. Affinity of AXIN1 to β-catenin is increased by GSK3β-mediated phosphorylation [29,30]. In the destruction complex, β-catenin is first phosphorylated by CK1α at Ser45, then by a serine/threonine kinase GSK3β at Ser 33, Ser 37 and Thr41. Interaction of GSK3β with β-catenin is facilitated by AXIN1 and APC [29,31]. F-box containing protein E3 ubiquitin ligases such as β-transducin repeat-containing protein (β-TrCP) marks β-catenin for ubiquitination and proteasomal degradation [4,22,23,32].

Upon binding of WNT proteins to the seven-pass transmembrane frizzled (FZD) receptor and single pass transmembrane receptors LRP5 and LRP6, the initiation of canonical signaling pathway occurs, leading to β-catenin stabilization (Figure 1B) [7,32]. A characteristic feature of FZD is the cysteine-rich domain, which is the primary module for binding of WNT ligands [4,13]. The mutual interaction of WNT ligands with both FZD and LRP5/6 is necessary for canonical pathway activation that inhibits β-catenin proteasomal degradation [7,22,32]. It is preceded by a series of events, and several models have been created [33] to show the sequence of events leading to activation of β-catenin. Regardless of the model, disheveled (DVL) that directly interacts with FZD is concomitantly phosphorylated by several protein kinases such as protease-activated receptor 1 (PAR1), casein kinase 1ε (CK1ε), metastasis associated kinase (MAK) and protein kinase C (PKC) [14,34]. Activated DVL detaches AXIN1 from the destruction complex and the released AXIN1 binds to phosphorylated LRP5/6 [7,32]. Phosphorylation of LRP5 and LRP6 occurs in Pro-Pro-Pro(SerTrp)Pro (PPP(S/T)P) motifs and is triggered by GSK3β and CK1γ [23,34]. β-catenin can be dephosphorylated by protein phosphatase 2A (PP2A) [35]. WNT/β-catenin-signaling can be potentiated by the leucine-rich repeat-containing G-protein coupled receptor 5/a roof plate-specific spondin (LGR5/RSPO) complex, which acts in cooperation with receptors FZD/LRP5/6 [36,37,38]. The LRG5/RSPO complex promotes WNT-signaling through the neutralization ring finger protein 43 (RNF43) and zinc and ring finger protein 3 (ZNRF3), the transmembrane E3 ligases that serve as a part of a negative feedback loop [39]. All these processes lead to the accumulation of stable, unphosphorylated β-catenin in the cytoplasm, followed by its translocation to the nucleus. β-catenin binds to nucleoporins (NUPs) and builds the nuclear pore complex (NPC). It binds NUP358 on the cytoplasmic side, NUP62 in a central channel and NUP98 together with NUP153 on a nuclear end [40]. In the nucleus, after displacing the transcriptional repressor Groucho, β-catenin can interact with several proteins, e.g., T cell factor/lymphoid enhancer-binding factor 1 (TCF/LEF), brahma-related gene-1 (BRG-1), TATA-binding protein, CREB-binding protein/its homolog p300 (CBP/p300), c-JUN, SWItch/sucrose non-fermentable chromatin-remodeling complex (SWI/SNF) and B-cell CLL/lymphoma 9 protein (BCL-9; BCL9L), which links the N terminal part of β-catenin with pygopus (PYGO) [34,41]. Then, β-catenin serves as a transcriptional regulator of the expression of WNT target genes [7,21,32,34]. These genes encode the following groups of proteins: (a) regulators of proliferation, e.g., vascular endothelial growth factor (VEGF), fibroblast growth factor (FGF), c-JUN, FOS-related antigen 1 (FRA1); (b) regulators of the canonical WNT pathway e.g., WNT1-inducible-signaling pathway protein 1 (WISP1), AXIN, Dickkopf-1 (DKK1), TCF, LEF1; (c) matrix metalloproteinases and some components of extracellular matrix; (d) cadherins; (e) lineage-specific proteins such as microphthalmia-associated transcription factor (MITF), which modulates several functions in melanocytes and melanoma [42].

It should be noted that the transcriptional role of β-catenin extends beyond the TCF/LEF as β-catenin may be a partner of other transcription factors, e.g., sex-determining region Y (SRY) box-containing factors (SOX), mothers against decapentaplegic homolog (SMAD), octamer-binding transcription factor 4 (OCT4) and forkhead box class O family member proteins (FOXOs) [43].

WNT/β-catenin-signaling can be modulated by several antagonists at the ligand and receptor level, e.g., DKK proteins, secreted frizzled-related proteins (sFRPs), WNT inhibitory factor 1 (WIF1), WNT modulator in surface ectoderm (WISE), Kremen (KRM) and Cerberus protein (CER1) [14,16,44]. They interact with WNTs and their receptors, causing the inhibition of the WNT/β-catenin pathway [16]. Moreover, the activity of WNTs is also modulated by a highly conserved feedback antagonist NOTUM, acting as a deacylase that removes a palmitate moiety from WNTs leading to their inactivation [4]. There are several negative feedback mechanisms that can limit WNT-signaling, including WNT target genes such as *AXIN1, AXIN2, DKK1* and *SFRP* [45,46].

The β-catenin-dependent WNT-signaling cascade (Figure 1) regulates a wide range of biologic processes comprising both developmental processes during embryogenesis as well as those during tissue homeostasis and regeneration. Canonical WNT-signaling is involved in the regulation of cell proliferation and differentiation and maintenance of stem cells. β-catenin, the central component of WNT/β-catenin pathway is a multifunctional protein that can either bind to cadherin that is an integral part of the actin cytoskeleton or act as transcriptional coactivator [42,47]. β-catenin is expressed both in melanocytes and epithelium, however, the amount of β-catenin at the cell surface is less abundant in melanocytes than in epithelial cells [48]. β-catenin (781 amino acids) is encoded by *CTNNB1* and comprises a flexible N-terminal domain (NTD; ~150 aa), central armadillo (ARM) repeat domain (12 copies, 550 aa) and a C-terminal transactivation domain (CTD; ~100 aa) [23]. While NTD and CTD may be flexible, the central region forms a rigid scaffold that is responsible for the interaction of β-catenin with its binding partners [49] such as cytoskeletal proteins α-catenin, IQ motif containing GTPase activating protein 1(IQGAP1) [50], E-cadherin and N-cadherin [51], a conserved nuclear protein, named chibby, transcriptional regulators TCF/LEF, inhibitor of β-catenin and TCF4 (ICAT) and proteins forming the ‘destruction complex’.

### 1.3. Non-Canonical WNT Signaling

β-catenin-independent pathways comprise: WNT/planar cell polarity-signaling pathway (PCP) (Figure 2A) and WNT/Ca^2+^-signaling pathway (Figure 2B). The WNT/PCP-signaling pathway maintains planar cell polarity, as it is involved in regulation of modification of actin cytoskeleton structures and cell motility. It is activated by binding of WNT5A, WNT7A and WNT11 ligands to non-canonical FZD receptors along with tyrosine kinase co-receptors: protein tyrosine kinase 7 (PTK7), RAR-related orphan receptor (ROR) and receptor like tyrosine kinase (RYK) [52]. WNT5A is a key regulator of non-canonical WNT-signaling, however, it can play diverse roles in different types of cells, including tumor cells. Different roles of WNT5A, especially in various cancers, are partially due to the existence of two isoforms of WNT5A, WNT5A-long and WNT5-short [53]. Activation of DVL and further formation of the disheveled associated activator of morphogenesis 1 (DVL-Daam-1) complex activates the RHO GTPase that leads to activation of the RHO-associated kinase (ROCK) followed by modification of actin cytoskeleton and cytoskeletal rearrangement. DVL also activates the RAC GTPase that stimulates c-Jun N-terminal kinases (JNK) activity, which in turn positively regulates activator protein 1 (AP-1)-dependent genes. This network is involved in the modification of actin cytoskeleton structures influencing the polarization and motility of cells [17,54].

The WNT/Ca^2+^-signaling pathway is associated with the release of Ca^2+^ from intracellular stores. Interaction of WNT ligands with FZD activates phospholipase C (PLC) that hydrolyzes phosphatidylinositol (4,5)-biphosphates (PIP2) to inositol (1,4,5)-triphosphates (IP3) and diacylglycerol (DAG). DAG activates PKC kinase that in turn activates the small GTPase CDC42, while IP3 induces the release of Ca^2+^ ions from intracellular depots. Release of Ca^2+^ activates Ca^2+^/calmodulin dependent kinase II (CaMKII) and calcineurin (CaN). CaMKII phosphorylates TGFβ-activated kinase 1 (TAK1), which induces Nemo-like kinase (NLK) activation, which in turn inhibits transcriptional activity of canonical WNT-signaling. CaN via dephosphorylation activates nuclear factor of activated T-cells (NFAT) that translocates to the nucleus and regulates the expression of target genes. WNT/Ca^2+^ pathway activation plays an important role in the regulation of cell motility and cytoskeleton organization [17,54,55].

## 2. WNT Signaling in Cancer

As demonstrated in numerous studies, the aberrant activation of WNT-signaling contributes to malignant cell transformation and neoplastic proliferation with further metastatic dissemination and resistance to treatment [43,56]. Many surface markers of cancer stem cells (CSCs) (CD44, CD24, CD133, LGR5/GPR49, ABC cassette genes, EpCAM) are direct targets of the WNT pathway. CSCs provide the long-term maintenance of the tumor and contribute to poor clinical outcome of therapies [43]. Different genetic alterations can cause the inhibition of proteasomal degradation of β-catenin, resulting in the hyperactivation of canonical WNT-signaling and enhanced nuclear β-catenin accumulation. Genetic and epigenetic alterations affecting constituents of WNT pathways are tissue specific and they differ in frequency between cancers [56]. Apart from the most frequent mutations of *APC* in colorectal cancer and *CTNNB1* in hepatocellular carcinoma, deregulations of several extracellular modulators of WNT-signaling e.g., DKKs, sFRPs and WIF1 also contribute to cancer development. These proteins antagonize canonical WNT-signaling by binding to LRP5/6 or inhibit the interaction between WNTs and their receptors [6]. Moreover, vacuolar H^+^-ATPase (v-ATPase), an electrogenic H^+^ transporter required for WNT-signaling activation may also trigger abnormal WNT/β-catenin-signaling and contribute to WNT-signaling-dependent tumorigenesis. High expression of v-ATPase subunits has been observed in colorectal, prostate, breast, ovarian and pancreatic cancer cells [23]. Furthermore, it has also been demonstrated that β-catenin modulates the cancer microenvironment, participating in creating the niche for cancer progression [57]. The pre-metastatic niche permits both the implantation of tumor cells into distant organs as well as their survival [58,59,60,61]. However, high levels of nuclear β-catenin do not always indicate poor prognosis [57]. For that reason, it is necessary to consider the cell type-specific background in order to assess and understand the cellular outcome of aberrations in WNT-signaling [62].

## 3. WNT Signaling in Melanoma

### 3.1. Genetic and Epigenetic Alterations

Deregulations in the canonical WNT-signaling in cancer may result from diverse mutations and epigenetic mechanisms. Mutations in genes encoding distinct elements of the WNT pathway can cause (i) loss of function of the destruction complex, (ii) increase in nuclear localization of β-catenin resulting in β-catenin-mediated transactivation of several genes. Mutations are mostly detected in genes encoding components of the destruction complex such as *APC* and *AXIN*, but the gene encoding β-catenin, *CTNNB1*, is also frequently mutated in selected cancer types. High frequency of mutations causing hyperactivation of WNT-signaling can be detected for example in colorectal cancer [63,64] and mutations in *APC* leading to APC loss of function have been found in about 70% of cases [65,66,67].

In melanoma, frequencies of mutations in *APC*, *AXIN1* and *CTNNB1* are low, reaching according to cbioportal.org 10%, 2.9% and 5.9%, respectively (Table 1), however, a significant interstudy variability exists. An early study reported mutations in *CTNNB1* in six of 26 melanoma cell lines, and all these mutations affect phosphorylation of β-catenin rending it resistant to proteasomal degradation [68]. Several later studies have shown much lower frequencies of mutations in *CTNNB1* and *APC*, which put into question the importance of a genetic component in aberrant activity of β-catenin in melanoma [69,70,71,72,73]. For example, only 1 of 65 primary melanomas harbored mutations of *CTNNB1*, and one third of cases showed nuclear accumulation of β-catenin [69]. In another study, while a *CTNNB1* missense mutation and a truncating *APC* mutation were reported only in one out of forty cell lines, it has been demonstrated that hypermethylation of *APC* promoter was present in about 15% of melanoma biopsies and cell lines suggesting transcriptional silencing [74]. This epigenetic regulation was, however, not accompanied with increased WNT-signaling probably due to residual activity of APC. Epigenetic regulation of WNT/β-catenin antagonists such as DKKs, WIF1 and sFRP2 contributes substantially to activation of β-catenin. *DKK1*, *DKK2* and *DKK3* downregulation has been observed in melanoma cell lines and tissue samples, however, in contrast to other cancer types promoter hypermethylation is responsible only for downregulation of *DKK2* [75]. Promoter methylation of *WIF1* has been shown as leading to *WIF1* silencing [76,77]. Luo et al. have found that methylation of the *sFRP2* promoter results in a significant decrease of sFRP2 in patient melanoma samples and melanoma cell lines compared with the paired adjacent non-tumor tissue and non-transformed melanocytes, respectively [78].

### 3.2. Canonical Signaling

WNT-signaling plays an important function in skin. WNT/β -catenin-signaling guides the migration of neural crest cells, multipotent precursor cells and drives them toward a melanocyte fate [82,83,84], including terminal differentiation of melanoblasts to melanocytes [83]. WNT/β-catenin-signaling is also responsible for the maintenance of homeostasis between melanocytes and keratinocytes in normal epidermis [44,54]. The activation of MITF, a lineage-specific transcription factor, by the canonical WNT-signaling plays an important role in melanocytes [85]. It has been observed that the fate of neural crest cells depends on MITF, as cells with low MITF level differentiate toward e.g., neurons, glial cells or cartilage, whereas cells with high MITF give rise to melanocytes [84]. In the context of melanocyte lineage, β-catenin not only controls expression of *MITF*, but also modulates its function via direct protein–protein interaction [86].

As melanoma cells utilize WNT/β-catenin-signaling for transformation and proliferation, whereas non-canonical signaling for metastasis [87], β-catenin does not fully comply with the definition of an oncogene in this cancer [88]. The exact role of WNT-signaling in melanoma initiation and progression remains highly controversial to date despite extensive studies. While an increasing level of nuclear β-catenin during cancer progression is characteristic for several cancer types including colorectal cancer and hepatocellular carcinoma [89,90,91,92], the level of nuclear β-catenin is decreased in melanoma during disease progression [62,93,94,95,96,97]. However, opposite results have been published as well [98,99,100]. β-catenin hyperactivation in melanoma is rarely caused by mutations of *CTNNB1* [101]. β-catenin has been shown to play a critical role in the early stages of melanocyte transformation [102,103]. The initiation of melanoma includes enhanced proliferation of cells together with suppression of senescence leading to abnormal growth of melanocytes [104]. Senescence is mediated by two main tumor suppressor pathways: INK4α/RB and ARF/p53 [105]. The *INK4α-ARF* (CDKN2α) locus encodes two proteins: p16^INK4a^ and p14(p19)^ARF^, regulating RB1 and p53 pathways, respectively. Inactivation of the genetic locus *INK4α-ARF* is important in overcoming the senescence barrier to oncogenesis [104]. In a variety of cancers, this locus is inactivated by mutations, whereas it has also been found that *p16^INK4a^* expression can be silenced by activated β-catenin [103]. Delmas et al. observed the ability of β-catenin to bypass senescence by silencing the *p16^INK4a^* through a conserved TCF/LEF site in its promoter [103]. Activation of β-catenin in melanoma is a consequence of binding of WNT1 and WNT3A to their receptors FZD1/7 and co-receptors LRP5/6 and ROR1. These two ligands are crucial for bypassing melanocyte senescence and inducing transformation of melanocytes [103,106]. While benign nevi have been found to be positive for nuclear β-catenin, loss of nuclear β-catenin during melanoma progression to metastases has been reported [62,94,107]. The importance of canonical WNT-signaling in melanoma initiation is mostly associated with β-catenin ability to regulate the expression of a wide range of genes of the melanocyte lineage, and its involvement in regulation of proliferation is most likely related to the activation of MITF expression [42,44,62,108,109,110]. The rheostat model and phenotype switching model of MITF function in melanoma [111,112,113] suggest that a very low MITF level or the absence of MITF results in cell senescence, a low level is characteristic for invasive/dedifferentiated melanoma cells, whereas a high level of MITF pushes cells first toward a highly proliferative phenotype and finally to differentiation. Therefore, MITF is considered to be a master regulator of ‘phenotype switching’ between proliferative and invasive states, contributing to the high plasticity of melanoma cells in response to changes in the tumor microenvironment, including response to treatment [82,114,115,116,117]. Moreover, MITF plays an important pro-survival role in melanoma cells [118]. Recently, a differentiation model of melanoma has been proposed, in which the level of MITF is one of crucial factors determining subtypes of melanoma exerting differential vulnerability to drug-induced stress [119]. In view of the correlation between β-catenin and MITF expression, WNT-signaling also affects melanoma plasticity [120]. In actively proliferating melanoma cells, nuclear β-catenin/LEF1 triggers the expression of MITF that in turn activates transcription of several genes, including genes encoding cell cycle regulators p16, p21 and cyclin dependent kinase 2 (CDK2, pigmentation-related proteins such tyrosinase, dopachrome tautomerase (DCT) and Melan-A and pro-survival factors e.g., BCL2 and BCL2A1 [42,118,121]. Active β-catenin-signaling leads to increased differentiation in benign lesions [62,117] as it disrupts the regulatory balance between PAX3, SOX10 and MITF towards terminal differentiation [83]. It has been found that β-catenin mediates the activation of POU domain of transcription factor BRN2 that plays a complex role in regulation of MITF [83,101]. BRN2 tends to be highly expressed in melanomas, which suggests that it can be a positive regulator of melanoma survival and proliferation [83]. It has been reported that depending on the cellular context, BRN2 can transcribe or repress MITF. It can drive MITF-mediated proliferation in the presence of oncogenic BRAF, whereas in the absence of mutated BRAF it can represses MITF promoting invasion [101]. Moreover, the proliferative MITF^high^ subpopulation of melanoma cells producing melanoma antigen recognized by T cells 1 (MART1) are less invasive, whereas when this differentiation antigen is lost melanoma cells acquire highly invasive properties [122]. Differential behavior of β-catenin in epithelial cancers and melanoma is associated with opposing features of epithelial cells and melanocytes [99]. Therefore, it has been strongly emphasized that these diverse effects of β-catenin in melanoma cells and epithelial-derived cancers may be connected with the activity of MITF [62,99]. Arozarena et al. have shown the correlation between the expression of β-catenin and MITF both in cell lines and melanoma biopsies [62]. When low β-catenin and highly invasive cells were treated with forskolin, an agent upregulating MITF, the complete loss of membrane blebs and F-actin cortex contraction was observed. In line with this, melanoma cells with high β-catenin and high MITF levels have been characterized with an elongated filopodia phenotype and MITF reduction significantly increased rounded blebbing cells [62]. The round shape morphology is regulated by phosphorylation of RHO/ROCK-mediated myosin light chain (MLC) that induces the contraction of the cortical actin meshwork, regulating bleb retraction of cells.

Aberrant expression of WNT/β-catenin antagonists (e.g., DKKs, WIF1, sFRPs) is common in melanoma and is associated with elevated β-catenin level. Downregulation of DKKs has been detected in melanoma cell lines and tissue samples [75]. Reduced level of WIF1 has been found to contribute to constitutive activation of canonical WNT-signaling in melanoma cells, and *WIF1* overexpression resulted in downregulation of WNT-signaling and suppression of melanoma cell proliferation [76]. Differences in *WIF1* expression have been observed between its level in the primary tumor and sentinel lymph node (SLN) metastases [77]. Similarly, decreased expression of sFRPs, leading to upregulated WNT/β-catenin-signaling is also common in melanoma, and sFRP1 has been shown as an appealing candidate for a tumor suppressor [123]. Reduction of sFRP2 enhances the canonical WNT-signaling, whereas demethylation of its promoter inhibits the nuclear retention of β-catenin in melanoma cells and suppresses invasion [78]. Kaur et al. have found that sFRP2 is expressed by aged fibroblasts that play an important role in melanoma progression and this expression is associated with melanoma metastasis, therapy resistance and poor outcomes in elderly patients [82]. sFRP2 decreases β-catenin, MITF and apurinic/apyrimidinic endonuclease 1 (APE1). APE1 is the redox effector involved in DNA repair, so its decrease can attenuate the response of melanoma cells to DNA damage induced by reactive oxygen species, making melanoma cells more resistant to vemurafenib [82].

### 3.3. Non-Canonical WNT Signaling

**Figure 3:**
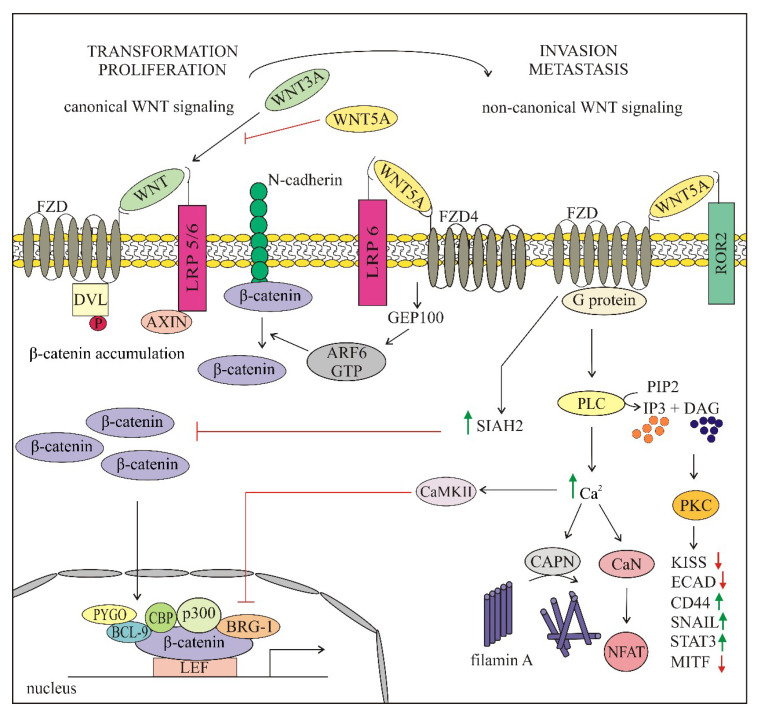
Proposed model of the crosstalk between canonical and non-canonical WNT-signaling in melanoma. In the canonical WNT pathway, WNT–FZD/LRP5/6 interaction initiates β-catenin dependent signaling. β-catenin translocates to the nucleus to drive the transcription of target genes. This is critical for early steps of transformation when melanocytes bypass senescence and start to proliferate, thus promoting first the radial then vertical growth of melanoma. An increase of WNT5A that activates non-canonical WNT-signaling inhibits β-catenin-signaling and enhances the invasiveness of melanoma cells crucial for metastatic spreading of melanoma. Green and red arrows indicate increase and decrease, respectively.

Non-canonical WNT-signaling, which is activated during melanoma progression, suppresses the WNT/β-catenin-signaling [107,124]. The interaction of canonical and non-canonical WNT-signaling in melanoma is presented in Figure 3. A lower level of nuclear β-catenin was detected in metastases than in primary melanoma in 343-melanoma samples [107]. Immunohistochemical analyses have revealed that most metastatic melanoma exhibited strong WNT5A staining comparing to benign tumors and a high WNT5A level in metastatic cutaneous melanoma is associated with poor outcome [125]. Therefore, it has been suggested that lack of nuclear β-catenin and high level of cytoplasmic WNT5A can be indicatives of unfavorable prognosis for melanoma patients [62,107,125]. Furthermore, inducible overexpression of WNT5A in melanoma cells exhibiting low metastatic activity results in enhanced invasiveness [126], whereas siRNA-mediated knockdown of ROR2, the WNT5A receptor, reduced the frequency and severity of lung metastases in mice [127]. Pro-metastatic effects of WNT5A have been associated with aerobic glycolysis promoted by WNT5A in melanoma cells [128]. WNT5A-mediated Ca^2+^-dependent release of exosomes by melanoma cells has been reported [129]. Exosomes are the smallest type of extracellular vesicles (30–180 nm in diameter), whose role in melanoma has been extensively discussed recently [61]. Using recombinant WNT5A in melanoma cells expressing low endogenous WNT5A induces a rapid release of exosomes containing the immunomodulatory cytokine interleukin 6 (IL-6) and the pro-angiogenic factors interleukin 8 (IL-8), VEGF and matrix metalloproteinase-2 (MMP2), whereas WNT5A depletion in melanoma cells expressing high endogenous WNT5A causes the reduction of IL-6, IL-8, VEGF and MMP2. Release of immunomodulatory and pro-angiogenic molecules enhances aggressiveness of melanoma cells and their capacity to metastasize [129]. Furthermore, it has been reported that WNT5A increases autophagy, which mediates resistance to various anticancer drugs. Knocking down autophagy-related gene 5 (ATG5) in WNT5A^high^ melanoma cells caused a reduction of the WNT5A level and induction of β-catenin [130].

WNT5A can either inhibit or activate canonical WNT-signaling in diverse ways depending on the receptor context [131]. Several of these mechanisms have been investigated in melanoma and the results revealed that WNT5A exerts a dichotomous role in melanoma (Figure 3). It can either stabilize β-catenin via adenosine diphosphate (ADP)-ribosylation factor 6 (ARF6) or suppress the WNT/β-catenin-signaling either by binding to FZD receptors or in a seven in absentia homolog 2 (SIAH2)-dependent manner. Binding of WNT5A to FZD4/LRP6 receptor complex activates ARF6 that in turn releases β-catenin from N-cadherin, which stimulates the shuttling of β-catenin between the membrane, cytoplasm and nucleus and enhances β-catenin-dependent transcription [132]. WNT5A that signals through FXD2, FZD5 and ROR2 activates PKC/Ca^2+^-signaling [87]. Calpain-1 (CAPN) mediates the cleavage of filamin A, promoting the remodeling of cytoskeleton and enhancing the motility of melanoma cells, whereas CAMKII inhibits β-catenin activity [87]. PKC activates signal transducer and activator of transcription 3 (STAT3) leading to inhibition of MITF expression with concomitant downregulation of melanocytic differentiation antigens (Melan A (MART1), DCT and gp100), thus promoting the metastatic phenotype [87]. Furthermore, PKC influences the motility of melanoma cells through the inhibition of metastasis repressor KISS-1 and E-cadherin and upregulation of metastasis-associated molecules, CD44 and SNAIL, resulting in transition to an invasive phenotype [87,133]. WNT5A can compete with WNT3A for binding to FZD2, and in this way LPR6 phosphorylation and β-catenin accumulation can be inhibited [122,134].

WNT5A can act through SIAH2 via the GSK3-β-independent pathway to promote degradation of β-catenin [135,136,137]. This mechanism of switching from WNT/β-catenin-signaling to non-canonical WNT-signaling has been associated with resistance to targeted therapy [136]. A high level of WNT5A promotes the resistance to vemurafenib, highlighting the opposing roles of different WNT pathways in melanoma [87]. Melanoma cells exerting a high level of WNT5A when exposed to vemurafenib treatment or other stress, developed a sort of drug resistant state (pseudosenescence), still retaining invasive capacity and the ability to form metastases. Furthermore, the pseudosenescent WNT5A-high cells were capable to express factors similar to those secreted by senescent fibroblasts and were positive for senescence markers, e.g., β-galactosidase, Src homology 2 domain containing F (SHF) and methylated of histone H3 on lysine 9 (H3K9Me). This WNT5A-mediated senescence-like response may be the mechanism that allows the tumor to evade therapy by undergoing growth arrest [122].

FZD3 has been found to be associated with β-catenin-independent-signaling and upregulation of this receptor is associated with melanoma progression and a reduced patient survival, whereas down-regulation of FZD3 suppresses growth and metastasis of melanoma [138]. Overexpressed FZD3 has been detected in 20% of melanoma patients whose tumors were deprived of infiltrating T cells indicating the importance of this receptor in immune evasion [139]. Moreover, it has been observed that FZD3 effectively modulates the activity of the mitogen-activated protein kinase/extracellular signal-regulated kinase (MAPK/ERK) pathway that is critical for melanoma maintenance. Therefore, FZD3 blocking agents may enhance the efficacy of melanoma treatment when used in combination with available kinase inhibitors and immunotherapy [138,139].

## 4. Crosstalk between WNT Signaling and Key Oncogenic Pathways Involved in Melanoma Development, Progression and Response to Treatment

**Figure 4:**
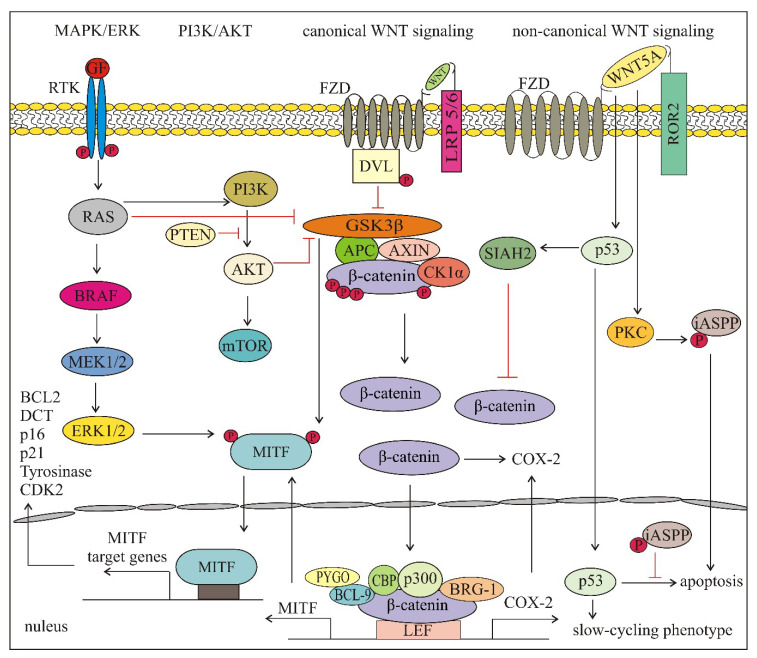
Main signal transduction pathways and transcriptional regulators that interact with the WNT-signaling pathways in melanoma. Figure shows the possible crosstalk between different pathways, however, some of the interactions are genetic context-, disease stage- or treatment-dependent. See the text for more details. APC—adenomatosis polyposis coli; AXIN—axis inhibition protein; BCL—B-cell CLL/lymphoma protein; BRG-1—brahma-related gene-1; CBP—(CREB)-binding protein; CK1α—casein kinase 1α; COX-2—cyclooxygenase 2; DVL—disheveled; FZD—frizzled receptor; GSK3β—glycogen synthase kinase 3β; iASPP—inhibitor of apoptosis-stimulating protein of p53; LEF—lymphoid enhancer-binding factor 1; LRP—low-density lipoprotein receptor related protein; MITF—microphthalmia-associated transcription factor; PTEN—phosphatase and tensin homolog deleted on chromosome ten; PYGO—pygopus; mTOR—mammalian target of rapamycin; PI3K—phosphatidylinositol-3-kinase; RAS—Rat sarcoma.; ROR—RAR-related orphan receptor; RTK—receptor tyrosine kinase—SIAH2—seven in absentia homolog 2.

The complex interactions existing between diverse signaling pathways create the opportunity for a cancer cell to compensate for a blockade of a single pathway. The crosstalk consisting of multiple interactions and feedback loops is crucial for the cellular response to microenvironmental changes, including treatment. WNT-signaling is an important part of the crosstalk between key oncogenic pathways involved in melanoma development, progression and response to treatment. Elements of the WNT-signaling pathways both depend on and regulate activities of diverse pathways such as MAPK/ERK and phosphatidylinositol-3-kinase (PI3K)/AKT as well as transcriptional regulators including p53 and MITF. Figure 4 shows main signal transduction pathways and transcriptional regulators that interact with the WNT-signaling pathways in melanoma.

WNT-signaling can interact with MAPK/ERK and PI3K/AKT-signaling in diverse ways. Moreover, growing evidence indicates that this crosstalk in melanoma depends on the genetic context and several mechanisms are considered as tumor stage-dependent or unique for melanoma. In an early study, it was shown that WNT/β-catenin and MAPK/ERK pathways synergized in order to induce melanoma without the need for p16INK4a mutations and β-catenin could cooperate with N-RAS [103]. Next, it was found that inhibition of BRAF^V600^ enhanced the activity of β-catenin in the presence of WNT ligands [140]. This combination of BRAF inhibition and WNT/β-catenin activation was accompanied by AXIN1 degradation and inhibition of GSK3β, whereas hyperactivated MAPK/ERK-signaling stabilized AXIN1 that inhibited WNT-signaling in melanoma [140]. This negative crosstalk between MAPK/ERK and WNT/β-catenin-signaling was considered as melanoma specific since in colon cancer and many other cancers, activation of WNT/β-catenin-signaling leads to the stabilization of both β-catenin and RAS and activation of MAPK-signaling [141,142]. In another study performed in a mouse model of melanoma and human biopsies, β-catenin stabilization was associated with increased activation of MAPK/ERK and PI3K/AKT-signaling in BRAF activated and PTEN inactivated highly metastatic melanomas [98]. Full activation of AKT was detected, however, only in a small subset of melanomas [98]. These results suggest that the crosstalk between MAPK/ERK, PI3K/AKT and WNT/β-catenin-signaling depends on the genetic context. When phenotypic effects of the WNT/β-catenin pathway were compared in wild-type and mutated PTEN melanomas, the invasiveness and bioenergetics of melanoma cells were found to be regulated by WNT3A/β-catenin in a PTEN-dependent manner [57]. A most recent report has demonstrated that there is a high variability in the activity of PI3K/AKT and WNT/β-catenin pathways in melanomas with BRAF^V600E^ and wild-type PTEN [143]. Moreover, the activity of these pathways can be largely modified in the response to targeted therapeutics and after the development of drug resistance [140,143,144]. Nuclear localization of β-catenin can be increased by prostaglandins, which indicates the potential crosstalk between WNT-signaling and the cyclooxygenase 2 (COX-2)/prostaglandin pathway [145]. COX-2 is a target gene of β-catenin transcription, and in addition, β-catenin contributes to the stabilization of COX-2 mRNA [145].

Transcription factors responding to diverse signal transduction pathways play a key role in integrating multiple signals to give rise to the optimal phenotype confronting the cancer developmental stage and microenvironmental insults [85]. MITF, a melanocyte and melanoma specific transcription factor is under control of many signaling pathways and active β-catenin contributes to its transcription [85]. MAPK/ERK, PI3K/AKT and WNT/β-catenin-signaling pathways are also able to post-translationally modify MITF [115]. ERK-mediated phosphorylation of MITF results in increased binding of p300/CBP [146] as well as proteasomal degradation [147,148]. It is worth to note that WNT/β-catenin, MAPK/ERK and PI3K/AKT-signaling can converge to regulate nuclear transport of MITF [149]. Phosphorylation of MITF by ERK1/2 primes MITF for phosphorylation by GSK3β [149], a kinase that is inhibited by both PI3K/AKT and WNT/β-catenin-signaling. This dual phosphorylation is an example how multiple signaling pathways can converge to control the activity of a specific transcription factor. MITF activity is also dependent on the non-canonical WNT pathway as PKC can activate STAT3 leading to inhibition of MITF expression with concomitant downregulation of melanocytic differentiation antigens (Melan A (MART1), DCT and gp100), thus promoting the metastatic phenotype [87,101].

The WNT5A impact on p53 activity is another example of the influence of non-canonical WNT-signaling on the transcription factor-mediated phenotype of melanoma cells. As shown most recently, a high expression of WNT5A is observed in wild-type p53 melanomas representing 80% of this cancer, and more important, WNT5A can promote a slow-cycling phenotype of melanoma cells by increasing the half-life of wild-type p53 [150]. WNT5A expression can be enhanced by aging or treatment of melanoma with targeted therapy or irradiation [82,121,151]. Following different types of stress, a slow-cycling phenotype is induced, which in turn may promote melanoma survival, plasticity and heterogeneity. However, WNT5A can stabilize p53, p53-induced apoptosis is inhibited, and melanoma cells are arrested. As p53 has been shown to inhibit WNT-signaling [152,153,154], a feedback loop WNT-p53-WNT that is possible in wild type p53 melanomas has been suggested [150]. A possible explanation is that PKC activated by WNT5A phosphorylates the inhibitor of apoptosis stimulating protein (iASPP) leading to its nuclear localization, which prevents p53-induced apoptosis [133,155]. As SIAH2 is a target of p53 [156,157], WNT5A-stabilized p53 may in turn increase SIAH2 expression and β-catenin degradation in melanoma [150]. Most recently, it has been shown that β-catenin can be a direct partner of SOX10 via protein–protein interactions, which reduces SOX10 protein level in melanoma cells [158]. These results suggest that SOX10 considered as functionally crucial for melanoma survival can be suppressed by WNT/β-catenin-signaling. Of note, SOX10 level has not been reduced by main targeted therapeutics used in melanoma treatment [117,158], and in selected vemurafenib- or trametinib-resistant melanoma cell lines has been even increased [143]. Interestingly, SOX10 expression in hepatocellular carcinoma has been shown to correlate with enhanced level of β-catenin, and active β-catenin forming a stable SOX10/TCF4/β-catenin complex is necessary for the oncogenic effects of SOX10 [159]. These examples illustrate how direct and indirect interactions of WNT-signaling with transcription factors can influence the melanoma cell phenotype.

## 5. WNT-Signaling in Cancer Immunity

**Figure 5:**
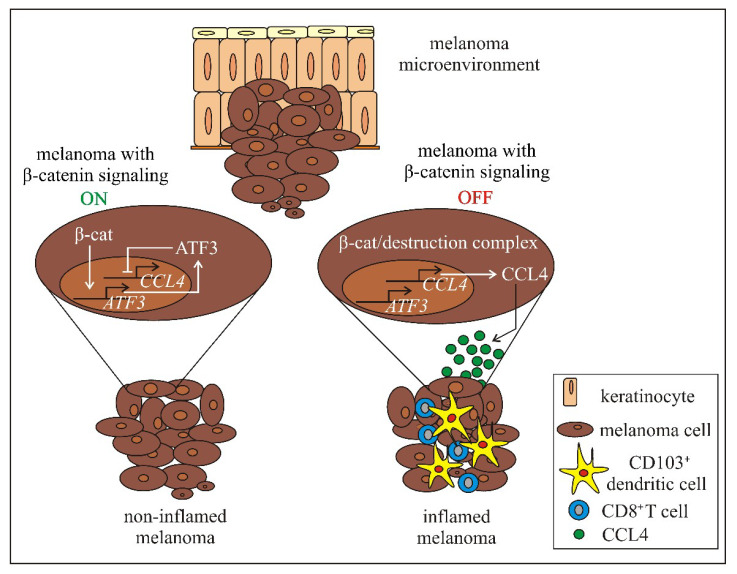
Mechanisms of immune exclusion in melanoma through WNT/β-catenin-signaling [172]. β-catenin induces expression of *ATF3* and ATF3 represses transcription of *CCL4*. CD103^+^ dendritic cells and cytotoxic CD8^+^ T-cells are not recruited to melanoma, which leads to non-inflamed tumor. When β-catenin-signaling is inactive in melanoma cells, *ATF3* is not expressed, which restores CCL4 production and secretion. This stimulates recruitment of immune cells, including CD103^+^ dendritic cells that activate CD8^+^ T cells. Recruitment of tumor specific CD8^+^ T cells in the tumor microenvironment results in immune inflamed melanoma. ATF3—activating transcription factor 3; β- cat—β- catenin; CCL4—CC-motif chemokine ligand 4.

Various studies indicate that WNT-signaling is associated with several aspects of immunity, and alterations in WNT-signaling can be connected with the deregulation of immune response against cancer, both innate and adaptive [56,160,161,162,163,164,165,166,167]. Tumors can be classified as having or lacking a T-cell-inflamed microenvironment [168] and immunotherapeutic interventions, including checkpoint inhibitors such as antiprogrammed death 1 (PD-1), programmed death-ligand 1 (PD-L1) and anti-cytotoxic T lymphocyte-associated antigen 4 (CTLA-4) antibodies have shown efficacy in patients with a preexisting T cell-inflamed cancer microenvironment [169]. Therefore, understanding the mechanisms underlying T cell exclusion is of clinical relevance. Most recently performed analysis of tumor microenvironment has revealed that about one-third of solid tumors have poor T cell infiltration, and tumor-intrinsic WNT/β-catenin-signaling inversely correlates with a T cell-inflamed phenotype in 90% of tumor types [170]. Authors suggest that activation of WNT/β-catenin in tumor cells is one of the possible mechanisms of intrinsic resistance to immunotherapy. The link between the WNT/β-catenin pathway and immune exclusion has been first identified in melanoma [171,172]. Gene expression profiling and exome sequencing of 266 individual melanoma metastases has revealed that about 48% of the non-inflamed melanomas exert active WNT/β-catenin pathway with elevated expression of genes under control of this signaling [171]. Mutations potentially leading to the WNT/β-catenin pathway activation, either gain-of-function mutations in *CTNNB1* (8%) or loss of function mutations (11%) in genes encoding negative regulators of WNT/β-catenin-signaling have been detected in these metastases [171]. Genetically engineered mouse models expressing conditionally active β-catenin have been used to show that melanomas with enhanced activation of WNT/β-catenin lack T cell infiltrate, which is due to insufficient recruitment of CD103/CD8α dendritic cells (DCs) [172]. This escape of immunity developed after activation of WNT-signaling in melanoma cells is accompanied by reduced secretion of CC-motif chemokine ligand 4 (CCL4), a chemokine that attracts the immune cells (Figure 5). The CCL4 expression is downregulated by β-catenin via activating transcription factor 3 (ATF3) [172]. Interestingly, no therapeutic effect of anti-CTLA-4 and anti-PD-L1 was observed in mice with melanoma expressing active β-catenin unless DCs were injected to activate tumor antigen-specific T cells. As immune evasion mediated by WNT/β-catenin-signaling frequently operates already in primary melanomas, it has been suggested that β-catenin-based stratification of patients may improve immunotherapeutic outcomes [173].

As activation of tumor-intrinsic WNT/β-catenin-signaling is enhanced in non-T cell-inflamed tumors, the WNT-signaling inhibitors may be useful for restoring immune cell infiltration to support immunotherapy.

## 6. WNT-Targeted Therapies

The important role of WNT-signaling in the development and adult life as well as its implication in a wide spectrum of diseases, including cancer has already attracted the attention of medical and biotech companies. Ongoing research is focused on the development of specific inhibitors of WNT/β-catenin-signaling for cancer therapies, and only very few modulators of non-canonical signaling are known. Based on the recent advances in cancer biology, targeting of WNT-signaling in cancer cells has to be put into perspective of its effectiveness either in reversing resistance to anticancer drugs or in inhibition of tumor evasion. It is important to underline that it is difficult to target only the WNT pathway without interfering with other signaling pathways. Diverse compounds of natural origin or synthetic, peptides and antibodies that are in preclinical studies are shown in Table 2. Natural compounds targeting WNT-signaling have been discussed extensively [174,175], and therefore only few of them are included in Table 2.

Several agents influencing WNT-signaling have reached the clinical trials (Table 3). Many of these inhibitors cause, however, severe side effects associated with impairment of tissue homeostasis and tissue regeneration, and off-target effects of WNT inhibitor are still an unresolved problem. Most of the clinical trials are conducted with patients with colorectal cancer (Table 3), and so far, melanoma patients were included only in one clinical trial (NCT01351103).

As shown in Table 2 and Table 3, WNT pathways can be affected by drugs at various stages. The first stage, WNT ligand activation and binding, is the most intensively investigated part of the WNT/β-catenin pathway. Porcupine, an enzyme with the acyl-transferase activity necessary for palmitoylation of WNT ligands, was one of the first targets [6,8,14]. The porcupine inhibitor WNT974 (also known as LGK974) is under clinical evaluation in several cancer types, including melanoma (NCT01351103) [184,185] (Table 3). C59 is another porcupine inhibitor investigated in melanoma that diminished WNT/β-catenin-signaling. It synergizes with anti-CLA-4 antibody in the B16 melanoma model, suggesting a synergistic enhancement in antitumor immunity [176]. A monoclonal antibody (WNT-2Ab) against human WNT2 ligand has been developed to induce apoptosis in melanoma cells exerting WNT2 overexpression, and WNT-2Ab treatment downregulated β-catenin target genes, e.g., CCND1 and c-Myc [177]. Phase 1 clinical trial for Foxy-5 (formylated 6-amino-acid peptide fragment), a WNT5A-mimicking peptide has already been completed in colorectal cancer and has shown a promising therapeutic value [191,192]. FZD receptors are potential targets of WNT directed therapies. The attenuation of WNT-signaling can be achieved by ubiquitylation-mediated degradation of FZD by anti-RSPO3 mAb (OMP-131R10). Phase 1 clinical trial employing OMP-131R10 has been completed with colorectal cancer patients [193]. OMP-18R5 (Vantictumab), a human monoclonal antibody has been used to inhibit ligand binding by targeting FZD receptors (FZD 1, 2, 5, 7, 8) [190]. The activity of OMP-54F28 (Ipafricept, completed phase I clinical trial) is based on the competition with FZD8 for ligand binding. OMP-54F28 is a recombinant fusion protein that consists of cysteine-rich domain of FZD8 and Fc domain of immunoglobulin and it functions as a trap for FZD8-binding of WNTs [189,206]. FZD can also be targeted by niclosamide, a plant derived compound that is capable of inducing FZD1 internalization and DVL down-regulation [194,207]. It downregulates B-cell lymphoma 9 (BCL9) that impairs the formation of active β-catenin/TCF/LEF triple-complex and upregulates c-JUN [200]. Niclosamide has already entered clinical trials in colorectal cancer. Poor systemic bioavailability of niclosamide led to the development of pro-drugs with better pharmacokinetic properties [208]. DKK1 inhibitors also belong to molecules targeting WNT-signaling, as DKK1, initially characterized as tumor suppressor may also function as tumor promoter [209]. DKN-01, a humanized monoclonal neutralizing antibody against DKK1 recognized as having potential therapeutic implications [210], has entered clinical trials. There are also WNT-signaling inhibitors that target FZD-DVL interaction e.g., FJ9 that disrupts the interaction between FZD7 and DVL. FJ9 has been found to downregulate canonical WNT-signaling and induce apoptosis in lung cancer and melanoma cells [178]. Another group of inhibitors belongs to intracellular drugs targeting the destruction complex. Tankyrases are the enzymes that degrade AXIN1 and AXIN2 through poly ADP-ribosylation. A very recent report indicates that inhibition of tankyrase by G007-LK can be used to overcome WNT/β-catenin-mediated resistance to immune checkpoint inhibitors [179]. While previously used tankyrase inhibitors caused bone loss [211] and intestinal toxicity [212], no such signs were observed in mice treated with G007-LK [179]. Growing evidence indicates that dietary factors along with alteration in the gutactivator protein 1 microbiota can affect WNT-signaling. Genistein, a soy-derived isoflavone, for which clinical trial phase 1 and 2 has been completed in CRC, inactivates WNT-signaling by GSK3-β targeting [202,203,213]. Fisetin (3,7,3′,4′-tetrahydroxyflavone) is a dietary flavonoid that inhibits GSK3-β and activates β-catenin in melanoma cells [180]. Another plant-derived molecule known to modulate WNT-signaling in melanoma is a triterpene lupeol, (lup-20(29)-en-3β-ol). It has been found that lupeol prevented the translocation of β-catenin to the nucleus, therefore decreased nuclear β-catenin level and expression of β-catenin target genes e.g., coding region determinant-binding protein (*CRD-BP*), *MITF* and *CCND1* [182]. Pentoxifylline, a drug approved by the FDA for the treatment of peripheral arterial disease, markedly reduced the level of active β-catenin in the nucleus of melanoma cells with high basal expression of β-catenin [183].

**Figure 6:**
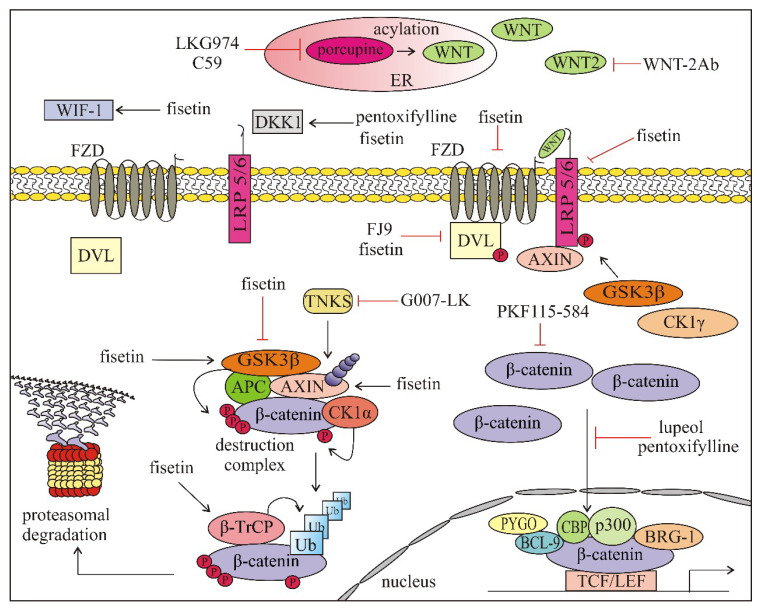
Compounds and antibodies affecting WNT/β-catenin-signaling pathway that were investigated in melanoma (preclinical studies, except for LGK974, which is tested in the clinical trial NCT01351103). APC—adenomatosis polyposis coli; AXIN—axis inhibition protein; BRG-1—brahma-related gene-1; BCL-9—B-cell CLL/lymphoma 9 protein; CBP—cAMP response element-binding protein; CK1α—casein kinase 1 α; CK1γ—casein kinase 1 γ; DKK1—Dickkopf-1; DVL—disheveled; ER—endoplasmic reticulum; FZD—frizzled; GSK3β—glycogen synthase kinase 3β; LRP5/6—lipoprotein receptor related protein 5/6; PYGO—pygopus; TKNS—tankyrase; β-TrCP—beta-transducin repeat–containing protein; Ub—ubiquitin.

As demonstrated above, there is a large group of natural or synthetic compounds, peptides and antibodies that are capable of affecting, either inducing or inhibiting, the WNT-signaling pathways at various stages. Compounds that have been investigated in melanoma are shown in Figure 6.

While inhibition of WNT-signaling represents an immensely appealing strategy for the development of anticancer therapeutics, none of drugs targeting WNT-signaling is yet available in clinical practice [4,214].

## 7. Conclusions

WNT-signaling is extremely complex and context-dependent in cancer, including melanoma. Deregulation of WNT-signaling contributes to cancer initiation, progression, modulation of immune microenvironment and resistance to treatment. While WNT-signaling alterations start with *APC* mutations in about 70% of colorectal cancer patients, mutations are not the major cause of these deregulations in melanoma. Moreover, transcriptionally active β-catenin is associated with less invasive disease and more favorable prognosis for melanoma patients, in contrast to other cancers, in which nuclear β-catenin is a driving force of both initiation and progression. It is thought that β-catenin-suppressed invasion occurs through a cell-type specific mechanism involving transcription factor MITF, one of the β-catenin target genes. On the other hand, low efficacy of immunotherapy is observed in melanomas with elevated level of β-catenin. The identification of therapeutic targets is further complicated by the crosstalk between WNT-signaling pathways and other signaling pathways crucial for melanoma development such as MAPK/ERK and PI3K/AKT, as well as the plasticity of melanoma cells in response to microenvironmental insults. Therefore, finding a therapeutic window for effective modulation of the WNT pathway in melanoma is a challenging task. A low number of clinical trials investigating WNT/β-catenin modulators in melanoma patients is the consequence of the controversial role of WNT-signaling in melanoma.

## Figures and Tables

**Table 1:** Frequency of mutations of genes encoding elements of the canonical WNT-signaling pathway detected in melanoma.

Gene	Literature Data		Melanoma (cbioportal.org; 1284 Cases)
*APC*	15%	[74]	10.0%
	11%	[79]	
	2.7%	[73]	
*AXIN1*	–		2.9%
*AXIN2*	11%	[79]	1.3%
*BCL9*	–		5.7%
*CTNNB1*	23%	[68]	5.9%
	4.6%	[71]	
	3.3%	[71]	
	11%	[79]	
	1.6%	[72]	
	1.5%	[69]	
	5.4%	[73]	
	3.2%	[70]	
	3.7%	[70]	
*FBXW7*	8.1%	[80]	3.4%
	8.3%	[81]	
*GSK3β*	–		1.2%
*SFRP*	–		1.7%
*WIF1*	–		2.8%

**Table 2:** WNT/β -catenin pathway inhibitors in melanoma (preclinical study).

Compound	Target Mode of Action	Research Model	References
C59	porcupine inhibitor (synergizes with CTLA4-targeting antibodies in mouse melanoma models)	WM266 human melanoma cell line; B16/F10 mouse melanoma cell line; patient derived Hu-175, Hu-422, Hu-424, Hu-451 human melanoma cell lines	[176]
WNT-2Ab	WNT2 antibody	LOX, FEMX melanoma cell lines;	[177]
FJ9	DVL inhibitor	LOX melanoma cell line	[178]
G007-LK	tankyrase inhibitor (sensitizes melanoma to PD-1 immune checkpoint blockade)	B16/F10 mouse melanoma cell line	[179]
fisetin	(i) GSK3-β inhibitor	B16F10 mouse melanoma cell line	[180]
	(ii) activator of GSK3-β, DKK1 and WIF-1; FZD and LRP5/6 inhibitor	451Lu human melanoma cells; athymic (*nu/nu*) female nude mice	[181]
lupeol	blocking the translocation of β-catenin to the nucleus	Mel 928, Mel 1241 and Mel 1011 human melanoma cell lines	[182]
pentoxifylline	β-catenin	DMBC11, 12, 17, 19, 21 patient-derived melanoma cell lines	[183]
PKF115-584	β-catenin	SKMEL28, A375, BLM, SKMEL19 and 451Lu human melanoma cell lines	[100]

**Table 3:** Overview of clinical trials (current and past clinical trials) evaluating activity of drugs targeting WNT pathway in melanoma, colorectal cancer and hepatocellular carcinoma (https://clinicaltrials.gov).

Compound	Company	Target/Mode of Action	Type of Cancer	Trial Identifier (phase/status)	Ref.
WNT974 (with LGX818 & cetuximab)	Array BioPharma	porcupine inhibitor	metastatic colorectal cancer	NCT02278133 (Phase 1; Phase 2/completed; updated: October 2017)	[184,185]
LGK974 (monotherapy or combined with PDR001)	Novartis Pharmaceuticals	porcupine inhibitor	BRAF mutant colorectal cancer & melanoma	NCT01351103 (Phase 1 /recruiting; updated: June 2020)	[184,185]
ETC-1922159 (with pembrolizumab)	EDDC, A*STAR Research Entities	porcupine inhibitor	colorectal cancer	NCT02521844 (Phase 1/active, not recruiting; updated: October 2019)	[186]
CGX1321	Curegenix, Inc.	porcupine inhibitor	colorectal adenocarcinoma hepatocellular carcinoma	NCT03507998 (Phase 1/ recruiting; updated: May 2019)	[187]
CGX1321 (with pembrolizumab)	Curegenix, Inc.	porcupine inhibitor	colorectal cancer	NCT02675946 (Phase1/ recruiting updated: September 2019)	[187]
PRI-724	Prism Pharma Co., Ltd.	interaction of β-catenin and CBP	colorectal cancer	NCT01302405 (Phase 1 terminated due to low enrollment, updated: August 2017)	[188]
DKN-01 (monotherapy or combined with sorafenib)	Johannes Gutenberg University Mainz	DKK1 inhibitor	hepatocellular carcinoma	NCT03645980 (Phase 1; Phase 2/recruiting; updated: August 2019)	none
OMP-54F28 (ipafricept) with sorafenib	OncoMed Pharmaceuticals, Inc.	FZD8 decoy receptor for WNT ligands	locally advanced or metastatic hepatocellular cancer solid tumors	NCT02069145 (Phase 1/completed; updated: August 2017)	[189]
OMP-54F28 (ipafricept)				NCT01608867 (Phase 1/completed; updated: July 2017)	
OMP-18R5 (vantictumab)	OncoMed Pharmaceuticals	FZD receptors (1, 2, 5, 7, 8) inhibitors	solid tumors	NCT01345201 (Phase 1/completed; updated: September 2016)	[190]
Foxy-5	WntResearch AB	WNT5A mimetic	colorectal cancer	NCT02655952 & NCT02020291 (Phase 1/completed; updated: December 2018) NCT03883802 (Phase 2/recruiting; updated: April 2019)	[191,192]
			Metastatic colorectal cancer		
OMP-131R10 (with FOLFIRI)	OncoMed Pharmaceuticals, Inc.	anti-R-spondin3 antibody	colorectal cancer	NCT02482441 (Phase 1/completed; updated: August 2018)	[193]
niclosamide	Michael Morse, MD	FZD1 internalization and BCL-9 inhibition, c-JUN upregulation	colorectal cancer	NCT02687009 (Phase 1/terminated, updated: February 2020)	[194,195,196,197,198,199,200]
	Charite University, Berlin, Germany		metastatic colorectal cancer	NCT02519582 (Phase 2 /recruiting; updated: September 2018)	[201]
genistein	Sofya Pintova	GSK3-β inhibitor	colorectal cancer	NCT01985763 (Phase 1; Phase 2/completed; updated: May 2019)	[202,203]
resveratrol	University of California, Irvine	β-catenin/TCF interaction	colorectal cancer	NCT00256334 (Phase 1/completed updated: June 2014)	[204]
curcumin (dietary supplements: Mirtoselect^®®®®^ & Meriva^®®®®^)	Ente Ospedaliero Ospedali Galliera	β-catenin/TCF interaction	colorectal adenoma	NCT01948661 (Phase not applicable, trial without FDA-defined phases /active, not recruiting; updated: August 2018)	[205]

EDDC, Experimental Drug Development Center.

## Abbreviations

AP-1	activator protein 1
APC	adenomatosis polyposis coli
APE1	apurinic/apyrimidinic endonuclease 1
ARF6	adenosine diphosphate (ADP)-ribosylation factor 6
ARM	armadillo
ATF3	activating transcription factor 3
ATG5	autophagy-related gene 5
AXIN	axis inhibition protein
B9 L	B-cell lymphoma 9-like
BCL-9	B-cell CLL/lymphoma 9 protein
β-TrCP	beta-transducin repeat–containing protein
BRG-1	brahma-related gene-1
CaMKII	Ca^2+^/calmodulin dependent kinase II
CaN	calcineurin
CAPN	calpain
CBP	(CREB)-binding protein
CCL4	CC-motif chemokine ligand 4
CDK2	cyclin dependent kinase 2
cer1	Cerberus protein
CK1 γ	casein kinase 1 γ
CK1α	casein kinase 1α
CK1ε	casein kinase 1ε
CRC	colorectal cancer
CSCs	cancer stem cells
CTD	C-terminal domain
CTLA-4	anti-cytotoxic T lymphocyte-associated antigen 4
Daam-1	disheveled associated activator of morphogenesis 1
DAG	diacylglycerol
DCs	dendritic cells
DCT	dopachrome tautomerase
DKK	Dickkopf
DVL	disheveled
ER	endoplasmic reticulum
EVI/WLS	Evenness interrupted/Wntless
FGF	fibroblast growth factor
FOXOs	forkhead box class O family member proteins
FRA1	FOS-related antigen 1
FZD	frizzled
GSK3β	glycogen synthase kinase 3β
H3K9me	methylation of histone H3 on lysine 9
HCC	hepatocellular carcinoma
iASPP	inhibitor of apoptosis-stimulating protein of p53
ICAT	inhibitor of β-catenin and TCF4
IL-β	interleukin-1β
IP3	inositol (1,4,5)-triphosphates
IQGAP1 IQ	IQ motif containing GTPase activating protein 1(IQGAP1)
JNK	c-Jun N-terminal kinases
KRM	Kremen
LGR	leucine-rich repeat-containing G-protein coupled receptor
LGR5/RSPO	G-protein coupled receptor 5/a roof plate-specific spondin
LPPs	lipoprotein particles
LRP5/6	lipoprotein receptor related protein 5/6
MAK	metastasis associated kinase
MART1	melanoma antigen recognized by T cells 1
MITF	microphthalmia-associated transcription factor
MLC	myosin light chain
MMP	matrix metalloproteinase
NFAT	nuclear factor of activated T-cells
NLK	Nemo-like kinase
NPC	nuclear pore complex
NTD	N-terminal domain
NUPs	nucleoporins
OCT4	octamer-binding transcription factor 4
PAR1	protease-activated receptor 1
PCP	planar cell polarity-signaling pathway
PD-L1	programmed death-ligand 1
PIP2	phosphatidylinositol (4,5)-biphosphates
PKC	protein kinase C
PLC	phospholipase C
PP2A	protein phosphatase 2A
PPP(S/T)P	Pro-Pro-Pro(SerTrp)Pro
PTK7	protein tyrosine kinase 7
PYGO	pygopus
RNF43	ring finger protein 43
ROCK	Rho-associated kinase
ROR	RAR-related orphan receptor
RSPO	R-spondin, roof plate-specific spondin
RTK	receptor tyrosine kinase
RYK	receptor like tyrosine kinase
sFRPs	secreted frizzled-related proteins
SHF	Src homology 2 domain containing F
SIAH2	seven in absentia homolog 2
SLN	sentinel lymph node
SMAD	mothers against decapentaplegic homolog
SRY	sex-determining region Y
STAT3	Signal Transducer Moreover, Activator Of Transcription 3.
SWI/SNF	SWItch/Sucrose non-fermentable chromatin-remodeling complex
TAK1	TGFβ-activated kinase 1
TCF/LEF	T cell factor/lymphoid enhancer-binding factor 1
TGF-β	transforming growth factor-β
v-ATPase	vacuolar H^+^-Adenosine Triphosphatase.
VEGF	vascular endothelial growth factor
WIF1	WNT inhibitory factor 1
WISE	WNT modulator in surface ectoderm
WISP1	WNT1-inducible-signaling pathway protein 1
ZNRF3	zinc and ring finger protein 3

## References

[1] Nüsslein-Volhard C., Wieschaus E. (1980). Mutations affecting segment number and polarity in Drosophila. Nature.

[2] Nusse R., Varmus H.E. (1982). Many tumors induced by the mouse mammary tumor virus contain a provirus integrated in the same region of the host genome. Cell.

[3] Nusse R., Van Ooyen A., Cox D., Fung Y.K., Varmus H. (1984). Mode of proviral activation of a putative mammary oncogene (int-1) on mouse chromosome 15. Nature.

[4] Nusse R., Clevers H. (2017). Wnt/β-Catenin Signaling, Disease, and Emerging Therapeutic Modalities. Cell.

[5] Clevers H. (2006). Wnt/beta-catenin signaling in development and disease. Cell.

[6] Clevers H., Nusse R. (2012). Wnt/β-catenin signaling and disease. Cell.

[7] Kahn M. (2014). Can we safely target the WNT pathway?. Nat. Rev. Drug Discov..

[8] Zhan T., Rindtorff N., Boutros M. (2017). Wnt signaling in cancer. Oncogene.

[9] Kumar A., Chalamalasetty R.B., Kennedy M.W., Thomas S., Inala S.N., Garriock R.J., Yamaguchi T.P. (2016). Zfp703 Is a Wnt/β-Catenin Feedback Suppressor Targeting the β-Catenin/Tcf1 Complex. Mol. Cell. Biol..

[10] Grumolato L., Liu G., Mong P., Mudbhary R., Biswas R., Arroyave R., Vijayakumar S., Economides A.N., Aaronson S.A. (2010). Canonical and noncanonical Wnts use a common mechanism to activate completely unrelated coreceptors. Genes Dev..

[11] Van Amerongen R., Mikels A., Nusse R. (2008). Alternative wnt signaling is initiated by distinct receptors. Sci. Signal..

[12] Amin N., Vincan E. (2012). The Wnt signaling pathways and cell adhesion. Front. Biosci..

[13] Pai S.G., Carneiro B.A., Mota J.M., Costa R., Leite C.A., Barroso-Sousa R., Kaplan J.B., Chae Y.K., Giles F.J. (2017). Wnt/beta-catenin pathway: Modulating anticancer immune response. J. Hematol. Oncol..

[14] Komiya Y., Habas R. (2008). Wnt signal transduction pathways. Organogenesis.

[15] Buechling T., Boutros M. (2011). Wnt signaling signaling at and above the receptor level. Curr. Top. Dev. Biol..

[16] O’Connell M.P., Weeraratna A.T. (2009). Hear the Wnt Ror: How melanoma cells adjust to changes in Wnt. Pigment Cell Melanoma Res..

[17] Ackers I., Malgor R. (2018). Interrelationship of canonical and non-canonical Wnt signalling pathways in chronic metabolic diseases. Diab. Vasc. Dis. Res..

[18] Mirabelli C.K., Nusse R., Tuveson D.A., Williams B.O. (2019). Perspectives on the role of Wnt biology in cancer. Sci. Signal..

[19] Corbett L., Mann J., Mann D.A. (2015). Non-Canonical Wnt Predominates in Activated Rat Hepatic Stellate Cells, Influencing HSC Survival and Paracrine Stimulation of Kupffer Cells. PLoS ONE.

[20] Anagnostou S.H., Shepherd P.R. (2008). Glucose induces an autocrine activation of the Wnt/beta-catenin pathway in macrophage cell lines. Biochem. J..

[21] Tarapore R.S., Siddiqui I.A., Mukhtar H. (2012). Modulation of Wnt/β-catenin signaling pathway by bioactive food components. Carcinogenesis.

[22] Van Dyke T., Merlino G. (2012). β-catenin in metastatic melanoma—The smoking gun reloaded. Pigment Cell Melanoma Res..

[23] Jung Y.S., Park J.I. (2020). Wnt signaling in cancer: Therapeutic targeting of Wnt signaling beyond β-catenin and the destruction complex. Exp. Mol. Med..

[24] Liu C., Li Y., Semenov M., Han C., Baeg G.H., Tan Y., Zhang Z., Lin X., He X. (2002). Control of beta-catenin phosphorylation/degradation by a dual-kinase mechanism. Cell.

[25] Lee E., Salic A., Krüger R., Heinrich R., Kirschner M.W. (2003). The roles of APC and Axin derived from experimental and theoretical analysis of the Wnt pathway. PLoS Biol..

[26] Kim M.J., Chia I.V., Costantini F. (2008). SUMOylation target sites at the C terminus protect Axin from ubiquitination and confer protein stability. FASEB J..

[27] Mao J., Wang J., Liu B., Pan W., Farr G.H., Flynn C., Yuan H., Takada S., Kimelman D., Li L. (2001). Low-density lipoprotein receptor-related protein-5 binds to Axin and regulates the canonical Wnt signaling pathway. Mol. Cell.

[28] Huang S.M., Mishina Y.M., Liu S., Cheung A., Stegmeier F., Michaud G.A., Charlat O., Wiellette E., Zhang Y., Wiessner S. (2009). Tankyrase inhibition stabilizes axin and antagonizes Wnt signalling. Nature.

[29] Kim S.E., Huang H., Zhao M., Zhang X., Zhang A., Semonov M.V., MacDonald B.T., Zhang X., Garcia Abreu J., Peng L. (2013). Wnt stabilization of β-catenin reveals principles for morphogen receptor-scaffold assemblies. Science.

[30] Willert K., Shibamoto S., Nusse R. (1999). Wnt-induced dephosphorylation of axin releases beta-catenin from the axin complex. Genes Dev..

[31] Ikeda S., Kishida S., Yamamoto H., Murai H., Koyama S., Kikuchi A. (1998). Axin, a negative regulator of the Wnt signaling pathway, forms a complex with GSK-3beta and beta-catenin and promotes GSK-3beta-dependent phosphorylation of beta-catenin. EMBO J..

[32] Han J.I., Na K.J., Murph M. (2011). Wnt/β-Catenin signaling pathway in canine skin melanoma and a possibility as a cancer model for human skin melanoma. Melanoma in the Clinic Diagnosis, Management and Complications of Malignancy.

[33] Li V.S., Ng S.S., Boersema P.J., Low T.Y., Karthaus W.R., Gerlach J.P., Mohammed S., Heck A.J., Maurice M.M., Mahmoudi T. (2012). Wnt signaling through inhibition of β-catenin degradation in an intact Axin1 complex. Cell.

[34] Uzdensky A.B., Demyanenko S.V., Bibov M.Y. (2013). Signal transduction in human cutaneous melanoma and target drugs. Curr. Cancer Drug Targets.

[35] Su Y., Fu C., Ishikawa S., Stella A., Kojima M., Shitoh K., Schreiber E.M., Day B.W., Liu B. (2008). APC is essential for targeting phosphorylated beta-catenin to the SCFbeta-TrCP ubiquitin ligase. Mol. Cell.

[36] Carmon K.S., Gong X., Lin Q., Thomas A., Liu Q. (2011). R-spondins function as ligands of the orphan receptors LGR4 and LGR5 to regulate Wnt/beta-catenin signaling. Proc. Natl. Acad. Sci. USA.

[37] De Lau W., Barker N., Low T.Y., Koo B.K., Li V.S., Teunissen H., Kujala P., Haegebarth A., Peters P.J., Van de Wetering M. (2011). Lgr5 homologues associate with Wnt receptors and mediate R-spondin signalling. Nature.

[38] Glinka A., Dolde C., Kirsch N., Huang Y.L., Kazanskaya O., Ingelfinger D., Boutros M., Cruciat C.M., Niehrs C. (2011). LGR4 and LGR5 are R-spondin receptors mediating Wnt/β-catenin and Wnt/PCP signalling. EMBO Rep..

[39] De Lau W., Peng W.C., Gros P., Clevers H. (2014). The R-spondin/Lgr5/Rnf43 module: Regulator of Wnt signal strength. Genes Dev..

[40] Jamieson C., Sharma M., Henderson B.R. (2014). Targeting the β-catenin nuclear transport pathway in cancer. Semin. Cancer Biol..

[41] Townsley F.M., Cliffe A., Bienz M. (2004). Pygopus and Legless target Armadillo/beta-catenin to the nucleus to enable its transcriptional co-activator function. Nat. Cell Biol..

[42] Widlund H.R., Horstmann M.A., Price E.R., Cui J., Lessnick S.L., Wu M., He X., Fisher D.E. (2002). Beta-catenin-induced melanoma growth requires the downstream target Microphthalmia-associated transcription factor. J. Cell Biol..

[43] Ring A., Kim Y.M., Kahn M. (2014). Wnt/catenin signaling in adult stem cell physiology and disease. Stem Cell Rev. Rep..

[44] Larue L., Delmas V. (2006). The WNT/Beta-catenin pathway in melanoma. Front. Biosci..

[45] Glinka A., Wu W., Delius H., Monaghan A.P., Blumenstock C., Niehrs C. (1998). Dickkopf-1 is a member of a new family of secreted proteins and functions in head induction. Nature.

[46] Logan C.Y., Nusse R. (2004). The Wnt signaling pathway in development and disease. Annu. Rev. Cell Dev. Biol..

[47] Cici D., Corrado A., Rotondo C., Cantatore F.P. (2019). Wnt Signaling and Biological Therapy in Rheumatoid Arthritis and Spondyloarthritis. Int. J. Mol. Sci..

[48] Aktary Z., Bertrand J.U., Larue L. (2016). The WNT-less wonder: WNT-independent β-catenin signaling. Pigment Cell Melanoma Res..

[49] Huber A.H., Weis W.I. (2001). The structure of the beta-catenin/E-cadherin complex and the molecular basis of diverse ligand recognition by beta-catenin. Cell.

[50] Valenta T., Hausmann G., Basler K. (2012). The many faces and functions of β-catenin. EMBO J..

[51] Loh C.Y., Chai J.Y., Tang T.F., Wong W.F., Sethi G., Shanmugam M.K., Chong P.P., Looi C.Y. (2019). The E-Cadherin and N-Cadherin Switch in Epithelial-to-Mesenchymal Transition: Signaling, Therapeutic Implications, and Challenges. Cells.

[52] Qian D., Jones C., Rzadzinska A., Mark S., Zhang X., Steel K.P., Dai X., Chen P. (2007). Wnt5a functions in planar cell polarity regulation in mice. Dev. Biol..

[53] Kumawat K., Gosens R. (2016). WNT-5A: Signaling and functions in health and disease. Cell. Mol. Life Sci..

[54] Kulikova K., Kibardin A., Gnuchev N.V., Georgiev G.P., Larin S. (2012). Wnt signaling pathway and its significance for melanoma development. CTM Mod. Technol. Med..

[55] Lang C.M.R., Chan C.K., Veltri A., Lien W.H. (2019). Wnt signaling pathways in keratinocyte carcinomas. Cancers.

[56] Galluzzi L., Spranger S., Fuchs E., López-Soto A. (2019). WNT Signaling in Cancer Immunosurveillance. Trends Cell Biol..

[57] Brown K., Yang P., Salvador D., Kulikauskas R., Ruohola-Baker H., Robitaille A.M., Chien A.J., Moon R.T., Sherwood V. (2017). WNT/β-catenin signaling regulates mitochondrial activity to alter the oncogenic potential of melanoma in a PTEN-dependent manner. Oncogene.

[58] Kaplan R.N., Rafii S., Lyden D. (2006). Preparing the “soil”: The premetastatic niche. Cancer Res..

[59] Psaila B., Lyden D. (2009). The metastatic niche: Adapting the foreign soil. Nat. Rev. Cancer.

[60] Sceneay J., Smyth M.J., Möller A. (2013). The pre-metastatic niche: Finding common ground. Cancer Metastasis Rev..

[61] Gajos-Michniewicz A., Czyz M. (2019). Role of miRNAs in Melanoma Metastasis. Cancers.

[62] Arozarena I., Bischof H., Gilby D., Belloni B., Dummer R., Wellbrock C. (2011). In melanoma, beta-catenin is a suppressor of invasion. Oncogene.

[63] Schatoff E.M., Leach B.I., Dow L.E. (2017). Wnt Signaling and Colorectal Cancer. Curr. Colorectal Cancer Rep..

[64] White B.D., Chien A.J., Dawson D.W. (2012). Dysregulation of Wnt/β-catenin signaling in gastrointestinal cancers. Gastroenterology.

[65] Kwong L.N., Dove W.F. (2009). APC and its modifiers in colon cancer. Adv. Exp. Med. Biol..

[66] Rowan A.J., Lamlum H., Ilyas M., Wheeler J., Straub J., Papadopoulou A., Bicknell D., Bodmer W.F., Tomlinson I.P. (2000). APC mutations in sporadic colorectal tumors: A mutational “hotspot” and interdependence of the “two hits”. Proc. Natl. Acad. Sci. USA.

[67] Schell M.J., Yang M., Teer J.K., Lo F.Y., Madan A., Coppola D., Monteiro A.N., Nebozhyn M.V., Yue B., Loboda A. (2016). A multigene mutation classification of 468 colorectal cancers reveals a prognostic role for APC. Nat. Commun..

[68] Rubinfeld B., Robbins P., El-Gamil M., Albert I., Porfiri E., Polakis P. (1997). Stabilization of beta-catenin by genetic defects in melanoma cell lines. Science.

[69] Rimm D.L., Caca K., Hu G., Harrison F.B., Fearon E.R. (1999). Frequent nuclear/cytoplasmic localization of beta-catenin without exon 3 mutations in malignant melanoma. Am. J. Pathol..

[70] Omholt K., Platz A., Ringborg U., Hansson J. (2001). Cytoplasmic and nuclear accumulation of beta-catenin is rarely caused by CTNNB1 exon 3 mutations in cutaneous malignant melanoma. Int. J. Cancer.

[71] Demunter A., Libbrecht L., Degreef H., De Wolf-Peeters C., Van den Oord J.J. (2002). Loss of membranous expression of beta-catenin is associated with tumor progression in cutaneous melanoma and rarely caused by exon 3 mutations. Mod. Pathol..

[72] Pollock P.M., Hayward N. (2002). Mutations in exon 3 of the beta-catenin gene are rare in melanoma cell lines. Melanoma Res..

[73] Reifenberger J., Knobbe C.B., Wolter M., Blaschke B., Schulte K.W., Pietsch T., Ruzicka T., Reifenberger G. (2002). Molecular genetic analysis of malignant melanomas for aberrations of the WNT signaling pathway genes CTNNB1, APC, ICAT and BTRC. Int. J. Cancer.

[74] Worm J., Christensen C., Grønbaek K., Tulchinsky E., Guldberg P. (2004). Genetic and epigenetic alterations of the APC gene in malignant melanoma. Oncogene.

[75] Kuphal S., Lodermeyer S., Bataille F., Schuierer M., Hoang B.H., Bosserhoff A.K. (2006). Expression of Dickkopf genes is strongly reduced in malignant melanoma. Oncogene.

[76] Lin Y.C., You L., Xu Z., He B., Yang C.T., Chen J.K., Mikami I., Clément G., Shi Y., Kuchenbecker K. (2007). Wnt inhibitory factor-1 gene transfer inhibits melanoma cell growth. Hum. Gene Ther..

[77] Huynh K.T., Takei Y., Kuo C., Scolyer R.A., Murali R., Chong K., Takeshima L., Sim M.S., Morton D.L., Turner R.R. (2012). Aberrant hypermethylation in primary tumours and sentinel lymph node metastases in paediatric patients with cutaneous melanoma. Br. J. Dermatol..

[78] Luo X., Wei B., Chen A., Zhao H., Huang K., Chen J. (2016). Methylation-mediated loss of SFRP2 enhances melanoma cell invasion via Wnt signaling. Am. J. Transl. Res..

[79] Castiglia D., Bernardini S., Alvino E., Pagani E., De Luca N., Falcinelli S., Pacchiarotti A., Bonmassar E., Zambruno G., D’Atri S. (2008). Concomitant activation of Wnt pathway and loss of mismatch repair function in human melanoma. Genes Chromosomes Cancer.

[80] Aydin I.T., Melamed R.D., Adams S.J., Castillo-Martin M., Demir A., Bryk D., Brunner G., Cordon-Cardo C., Osman I., Rabadan R. (2014). FBXW7 mutations in melanoma and a new therapeutic paradigm. J. Natl. Cancer Inst..

[81] Balakrishnan A., Bleeker F.E., Lamba S., Rodolfo M., Daniotti M., Scarpa A., Van Tilborg A.A., Leenstra S., Zanon C., Bardelli A. (2007). Novel somatic and germline mutations in cancer candidate genes in glioblastoma, melanoma, and pancreatic carcinoma. Cancer Res..

[82] Kaur A., Webster M.R., Marchbank K., Behera R., Ndoye A., Kugel C.H., Dang V.M., Appleton J., O’Connell M.P., Cheng P. (2016). sFRP2 in the aged microenvironment drives melanoma metastasis and therapy resistance. Nature.

[83] Regad T. (2013). Molecular and cellular pathogenesis of melanoma initiation and progression. Cell. Mol. Life Sci..

[84] Mort R.L., Jackson I.J., Patton E.E. (2015). The melanocyte lineage in development and disease. Development.

[85] Vance K.W., Goding C.R. (2004). The transcription network regulating melanocyte development and melanoma. Pigment Cell Res..

[86] Schepsky A., Bruser K., Gunnarsson G.J., Goodall J., Hallsson J.H., Goding C.R., Steingrimsson E., Hecht A. (2006). The microphthalmia-associated transcription factor Mitf interacts with beta-catenin to determine target gene expression. Mol. Cell. Biol..

[87] Webster M.R., Kugel C.H., Weeraratna A.T. (2015). The Wnts of change: How Wnts regulate phenotype switching in melanoma. Biochim. Biophys. Acta.

[88] Lucero O.M., Dawson D.W., Moon R.T., Chien A.J. (2010). A re-evaluation of the “oncogenic” nature of Wnt/beta-catenin signaling in melanoma and other cancers. Curr. Oncol. Rep..

[89] Silva A.L., Dawson S.N., Arends M.J., Guttula K., Hall N., Cameron E.A., Huang T.H., Brenton J.D., Tavaré S., Bienz M. (2014). Boosting Wnt activity during colorectal cancer progression through selective hypermethylation of Wnt signaling antagonists. BMC Cancer.

[90] Metcalfe C., Ibrahim A.E., Graeb M., De la Roche M., Schwarz-Romond T., Fiedler M., Winton D.J., Corfield A., Bienz M. (2010). Dvl2 promotes intestinal length and neoplasia in the ApcMin mouse model for colorectal cancer. Cancer Res..

[91] Khalaf A.M., Fuentes D., Morshid A.I., Burke M.R., Kaseb A.O., Hassan M., Hazle J.D., Elsayes K.M. (2018). Role of Wnt/β-catenin signaling in hepatocellular carcinoma, pathogenesis, and clinical significance. J. Hepatocell. Carcinoma.

[92] Kim E., Lisby A., Ma C., Lo N., Ehmer U., Hayer K.E., Furth E.E., Viatour P. (2019). Promotion of growth factor signaling as a critical function of β-catenin during HCC progression. Nat. Commun..

[93] Kovacs D., Migliano E., Muscardin L., Silipo V., Catricalà C., Picardo M., Bellei B. (2016). The role of Wnt/β-catenin signaling pathway in melanoma epithelial-to-mesenchymal-like switching evidences from patients-derived cell lines. Oncotarget.

[94] Kageshita T., Hamby C.V., Ishihara T., Matsumoto K., Saida T., Ono T. (2001). Loss of beta-catenin expression associated with disease progression in malignant melanoma. Br. J. Dermatol..

[95] Maelandsmo G.M., Holm R., Nesland J.M., Fodstad Ø., Flørenes V.A. (2003). Reduced beta-catenin expression in the cytoplasm of advanced-stage superficial spreading malignant melanoma. Clin. Cancer Res..

[96] Bachmann I.M., Straume O., Puntervoll H.E., Kalvenes M.B., Akslen L.A. (2005). Importance of P-cadherin, beta-catenin, and Wnt5a/frizzled for progression of melanocytic tumors and prognosis in cutaneous melanoma. Clin. Cancer Res..

[97] Osborne J.E. (2002). Loss of beta-catenin expression is associated with disease progression in malignant melanoma. Br. J. Dermatol..

[98] Damsky W.E., Curley D.P., Santhanakrishnan M., Rosenbaum L.E., Platt J.T., Gould Rothberg B.E., Taketo M.M., Dankort D., Rimm D.L., McMahon M. (2011). β-catenin signaling controls metastasis in Braf-activated Pten-deficient melanomas. Cancer Cell.

[99] Gallagher S.J., Rambow F., Kumasaka M., Champeval D., Bellacosa A., Delmas V., Larue L. (2013). Beta-catenin inhibits melanocyte migration but induces melanoma metastasis. Oncogene.

[100] Sinnberg T., Levesque M.P., Krochmann J., Cheng P.F., Ikenberg K., Meraz-Torres F., Niessner H., Garbe C., Busch C. (2018). Wnt-signaling enhances neural crest migration of melanoma cells and induces an invasive phenotype. Mol. Cancer.

[101] Kaur A., Webster M.R., Weeraratna A.T. (2016). In the Wnt-er of life: Wnt signalling in melanoma and ageing. Br. J. Cancer.

[102] Larue L., Beermann F. (2007). Cutaneous melanoma in genetically modified animals. Pigment Cell Res..

[103] Delmas V., Beermann F., Martinozzi S., Carreira S., Ackermann J., Kumasaka M., Denat L., Goodall J., Luciani F., Viros A. (2007). Beta-catenin induces immortalization of melanocytes by suppressing p16INK4a expression and cooperates with N-Ras in melanoma development. Genes Dev..

[104] Larue L., Luciani F., Kumasaka M., Champeval D., Demirkan N., Bonaventure J., Delmas V. (2009). Bypassing melanocyte senescence by beta-catenin: A novel way to promote melanoma. Pathol. Biol..

[105] Collado M., Blasco M.A., Serrano M. (2007). Cellular senescence in cancer and aging. Cell.

[106] Goodall J., Martinozzi S., Dexter T.J., Champeval D., Carreira S., Larue L., Goding C.R. (2004). Brn-2 expression controls melanoma proliferation and is directly regulated by beta-catenin. Mol. Cell. Biol..

[107] Chien A.J., Moore E.C., Lonsdorf A.S., Kulikauskas R.M., Rothberg B.G., Berger A.J., Major M.B., Hwang S.T., Rimm D.L., Moon R.T. (2009). Activated Wnt/beta-catenin signaling in melanoma is associated with decreased proliferation in patient tumors and a murine melanoma model. Proc. Natl. Acad. Sci. USA.

[108] Hoek K.S., Eichhoff O.M., Schlegel N.C., Döbbeling U., Kobert N., Schaerer L., Hemmi S., Dummer R. (2008). In vivo switching of human melanoma cells between proliferative and invasive states. Cancer Res..

[109] Levy C., Khaled M., Fisher D.E. (2006). MITF: Master regulator of melanocyte development and melanoma oncogene. Trends Mol. Med..

[110] Kawakami A., Fisher D.E. (2017). The master role of microphthalmia-associated transcription factor in melanocyte and melanoma biology. Lab. Investig..

[111] Carreira S., Goodall J., Denat L., Rodriguez M., Nuciforo P., Hoek K.S., Testori A., Larue L., Goding C.R. (2006). Mitf regulation of Dia1 controls melanoma proliferation and invasiveness. Genes Dev..

[112] Hoek K.S., Goding C.R. (2010). Cancer stem cells versus phenotype-switching in melanoma. Pigment Cell Melanoma Res..

[113] Goding C.R. (2011). Commentary. A picture of Mitf in melanoma immortality. Oncogene.

[114] Wellbrock C., Arozarena I. (2015). Microphthalmia-associated transcription factor in melanoma development and MAP-kinase pathway targeted therapy. Pigment Cell Melanoma Res..

[115] Hartman M.L., Czyz M. (2015). MITF in melanoma: Mechanisms behind its expression and activity. Cell. Mol. Life Sci..

[116] Ahmed F., Haass N.K. (2018). Microenvironment-Driven Dynamic Heterogeneity and Phenotypic Plasticity as a Mechanism of Melanoma Therapy Resistance. Front. Oncol..

[117] Czyz M., Sztiller-Sikorska M., Gajos-Michniewicz A., Osrodek M., Hartman M.L. (2019). Plasticity of Drug-Naïve and Vemurafenib- or Trametinib-Resistant Melanoma Cells in Execution of Differentiation/Pigmentation Program. J. Oncol..

[118] Hartman M.L., Czyz M. (2015). Pro-survival role of MITF in melanoma. J. Investig. Dermatol..

[119] Tsoi J., Robert L., Paraiso K., Galvan C., Sheu K.M., Lay J., Wong D.J.L., Atefi M., Shirazi R., Wang X. (2018). Multi-stage Differentiation Defines Melanoma Subtypes with Differential Vulnerability to Drug-Induced Iron-Dependent Oxidative Stress. Cancer Cell.

[120] Arozarena I., Wellbrock C. (2017). Targeting invasive properties of melanoma cells. FEBS J..

[121] Carreira S., Goodall J., Aksan I., La Rocca S.A., Galibert M.D., Denat L., Larue L., Goding C.R. (2005). Mitf cooperates with Rb1 and activates p21Cip1 expression to regulate cell cycle progression. Nature.

[122] Webster M.R., Xu M., Kinzler K.A., Kaur A., Appleton J., O’Connell M.P., Marchbank K., Valiga A., Dang V.M., Perego M. (2015). Wnt5A promotes an adaptive, senescent-like stress response, while continuing to drive invasion in melanoma cells. Pigment Cell Melanoma Res..

[123] Mithani S.K., Smith I.M., Califano J.A. (2011). Use of integrative epigenetic and cytogenetic analyses to identify novel tumor-suppressor genes in malignant melanoma. Melanoma Res..

[124] Weeraratna A.T., Jiang Y., Hostetter G., Rosenblatt K., Duray P., Bittner M., Trent J.M. (2002). Wnt5a signaling directly affects cell motility and invasion of metastatic melanoma. Cancer Cell.

[125] Da Forno P.D., Pringle J.H., Hutchinson P., Osborn J., Huang Q., Potter L., Hancox R.A., Fletcher A., Saldanha G.S. (2008). WNT5A expression increases during melanoma progression and correlates with outcome. Clin. Cancer Res..

[126] Bittner M., Meltzer P., Chen Y., Jiang Y., Seftor E., Hendrix M., Radmacher M., Simon R., Yakhini Z., Ben-Dor A. (2000). Molecular classification of cutaneous malignant melanoma by gene expression profiling. Nature.

[127] O’Connell M.P., Fiori J.L., Xu M., Carter A.D., Frank B.P., Camilli T.C., French A.D., Dissanayake S.K., Indig F.E., Bernier M. (2010). The orphan tyrosine kinase receptor, ROR2, mediates Wnt5A signaling in metastatic melanoma. Oncogene.

[128] Sherwood V., Chaurasiya S.K., Ekström E.J., Guilmain W., Liu Q., Koeck T., Brown K., Hansson K., Agnarsdóttir M., Bergqvist M. (2014). WNT5A-mediated β-catenin-independent signalling is a novel regulator of cancer cell metabolism. Carcinogenesis.

[129] Ekström E.J., Bergenfelz C., Von Bülow V., Serifler F., Carlemalm E., Jönsson G., Andersson T., Leandersson K. (2014). WNT5A induces release of exosomes containing pro-angiogenic and immunosuppressive factors from malignant melanoma cells. Mol. Cancer.

[130] Ndoye A., Budina-Kolomets A., Kugel C.H., Webster M.R., Kaur A., Behera R., Rebecca V.W., Li L., Brafford P.A., Liu Q. (2017). ATG5 Mediates a Positive Feedback Loop between Wnt Signaling and Autophagy in Melanoma. Cancer Res..

[131] McDonald S.L., Silver A. (2009). The opposing roles of Wnt-5a in cancer. Br. J. Cancer.

[132] Grossmann A.H., Yoo J.H., Clancy J., Sorensen L.K., Sedgwick A., Tong Z., Ostanin K., Rogers A., Grossmann K.F., Tripp S.R. (2013). The small GTPase ARF6 stimulates β-catenin transcriptional activity during WNT5A-mediated melanoma invasion and metastasis. Sci. Signal..

[133] Dissanayake S.K., Wade M., Johnson C.E., O’Connell M.P., Leotlela P.D., French A.D., Shah K.V., Hewitt K.J., Rosenthal D.T., Indig F.E. (2007). The Wnt5A/protein kinase C pathway mediates motility in melanoma cells via the inhibition of metastasis suppressors and initiation of an epithelial to mesenchymal transition. J. Biol. Chem..

[134] Sato A., Yamamoto H., Sakane H., Koyama H., Kikuchi A. (2010). Wnt5a regulates distinct signalling pathways by binding to Frizzled2. EMBO J..

[135] Topol L., Jiang X., Choi H., Garrett-Beal L., Carolan P.J., Yang Y. (2003). Wnt-5a inhibits the canonical Wnt pathway by promoting GSK-3-independent beta-catenin degradation. J. Cell Biol..

[136] O’Connell M.P., Marchbank K., Webster M.R., Valiga A.A., Kaur A., Vultur A., Li L., Herlyn M., Villanueva J., Liu Q. (2013). Hypoxia induces phenotypic plasticity and therapy resistance in melanoma via the tyrosine kinase receptors ROR1 and ROR2. Cancer Discov..

[137] Webster M.R., Weeraratna A.T. (2013). A Wnt-er migration: The confusing role of β-catenin in melanoma metastasis. Sci. Signal..

[138] Li C., Nguyen V., Clark K.N., Zahed T., Sharkas S., Filipp F.V., Boiko A.D. (2019). Down-regulation of FZD3 receptor suppresses growth and metastasis of human melanoma independently of canonical WNT signaling. Proc. Natl. Acad. Sci. USA.

[139] Siemers N.O., Holloway J.L., Chang H., Chasalow S.D., Ross-MacDonald P.B., Voliva C.F., Szustakowski J.D. (2017). Genome-wide association analysis identifies genetic correlates of immune infiltrates in solid tumors. PLoS ONE.

[140] Biechele T.L., Kulikauskas R.M., Toroni R.A., Lucero O.M., Swift R.D., James R.G., Robin N.C., Dawson D.W., Moon R.T., Chien A.J. (2012). Wnt/β-catenin signaling and AXIN1 regulate apoptosis triggered by inhibition of the mutant kinase BRAFV600E in human melanoma. Sci. Signal..

[141] Guardavaccaro D., Clevers H. (2012). Wnt/β-catenin and MAPK signaling: Allies and enemies in different battlefields. Sci. Signal..

[142] Jeong W.J., Yoon J., Park J.C., Lee S.H., Lee S.H., Kaduwal S., Kim H., Yoon J.B., Choi K.Y. (2012). Ras stabilization through aberrant activation of Wnt/β-catenin signaling promotes intestinal tumorigenesis. Sci. Signal..

[143] Hartman M.L., Sztiller-Sikorska M., Gajos-Michniewicz A., Czyz M. (2020). Dissecting Mechanisms of Melanoma Resistance to BRAF and MEK Inhibitors Revealed Genetic and Non-Genetic Patient- and Drug-Specific Alterations and Remarkable Phenotypic Plasticity. Cells.

[144] Chien A.J., Haydu L.E., Biechele T.L., Kulikauskas R.M., Rizos H., Kefford R.F., Scolyer R.A., Moon R.T., Long G.V. (2014). Targeted BRAF inhibition impacts survival in melanoma patients with high levels of Wnt/β-catenin signaling. PLoS ONE.

[145] Serini S., Fasano E., Piccioni E., Monego G., Cittadini A.R., Celleno L., Ranelletti F.O., Calviello G. (2012). DHA induces apoptosis and differentiation in human melanoma cells in vitro: Involvement of HuR-mediated COX-2 mRNA stabilization and β-catenin nuclear translocation. Carcinogenesis.

[146] Price E.R., Ding H.F., Badalian T., Bhattacharya S., Takemoto C., Yao T.P., Hemesath T.J., Fisher D.E. (1998). Lineage-specific signaling in melanocytes. C-kit stimulation recruits p300/CBP to microphthalmia. J. Biol. Chem..

[147] Wu M., Hemesath T.J., Takemoto C.M., Horstmann M.A., Wells A.G., Price E.R., Fisher D.Z., Fisher D.E. (2000). c-Kit triggers dual phosphorylations, which couple activation and degradation of the essential melanocyte factor Mi. Genes Dev..

[148] Xu W., Gong L., Haddad M.M., Bischof O., Campisi J., Yeh E.T., Medrano E.E. (2000). Regulation of microphthalmia-associated transcription factor MITF protein levels by association with the ubiquitin-conjugating enzyme hUBC9. Exp. Cell Res..

[149] Ngeow K.C., Friedrichsen H.J., Li L., Zeng Z., Andrews S., Volpon L., Brunsdon H., Berridge G., Picaud S., Fischer R. (2018). BRAF/MAPK and GSK3 signaling converges to control MITF nuclear export. Proc. Natl. Acad. Sci. USA.

[150] Webster M.R., Fane M.E., Alicea G.M., Basu S., Kossenkov A.V., Marino G.E., Douglass S.M., Kaur A., Ecker B.L., Gnanapradeepan K. (2020). Paradoxical Role for Wild-Type p53 in Driving Therapy Resistance in Melanoma. Mol. Cell.

[151] Behera R., Kaur A., Webster M.R., Kim S., Ndoye A., Kugel C.H., Alicea G.M., Wang J., Ghosh K., Cheng P. (2017). Inhibition of Age-Related Therapy Resistance in Melanoma by Rosiglitazone-Mediated Induction of Klotho. Clin. Cancer Res..

[152] Levina E., Oren M., Ben-Ze’ev A. (2004). Downregulation of beta-catenin by p53 involves changes in the rate of beta-catenin phosphorylation and Axin dynamics. Oncogene.

[153] Liu J., Stevens J., Rote C.A., Yost H.J., Hu Y., Neufeld K.L., White R.L., Matsunami N. (2001). Siah-1 mediates a novel beta-catenin degradation pathway linking p53 to the adenomatous polyposis coli protein. Mol. Cell.

[154] Sadot E., Geiger B., Oren M., Ben-Ze’ev A. (2001). Down-regulation of beta-catenin by activated p53. Mol. Cell. Biol..

[155] Lu M., Breyssens H., Salter V., Zhong S., Hu Y., Baer C., Ratnayaka I., Sullivan A., Brown N.R., Endicott J. (2016). Restoring p53 Function in Human Melanoma Cells by Inhibiting MDM2 and Cyclin B1/CDK1-Phosphorylated Nuclear iASPP. Cancer Cell.

[156] Chan P., Möller A., Liu M.C., Sceneay J.E., Wong C.S., Waddell N., Huang K.T., Dobrovic A., Millar E.K., O’Toole S.A. (2011). The expression of the ubiquitin ligase SIAH2 (seven in absentia homolog 2) is mediated through gene copy number in breast cancer and is associated with a basal-like phenotype and p53 expression. Breast Cancer Res..

[157] Grishina I., Debus K., García-Limones C., Schneider C., Shresta A., García C., Calzado M.A., Schmitz M.L. (2012). SIAH-mediated ubiquitination and degradation of acetyl-transferases regulate the p53 response and protein acetylation. Biochim. Biophys. Acta.

[158] Uka R., Britschgi C., Krättli A., Matter C., Mihic D., Okoniewski M.J., Gualandi M., Stupp R., Cinelli P., Dummer R. (2020). Temporal activation of WNT/β-catenin signaling is sufficient to inhibit SOX10 expression and block melanoma growth. Oncogene.

[159] Zhou D., Bai F., Zhang X., Hu M., Zhao G., Zhao Z., Liu R. (2014). SOX10 is a novel oncogene in hepatocellular carcinoma through Wnt/β-catenin/TCF4 cascade. Tumour Biol..

[160] Hong Y., Manoharan I., Suryawanshi A., Majumdar T., Angus-Hill M.L., Koni P.A., Manicassamy B., Mellor A.L., Munn D.H., Manicassamy S. (2015). β-catenin promotes regulatory T-cell responses in tumors by inducing vitamin A metabolism in dendritic cells. Cancer Res..

[161] Alves C.H., Ober-Blöbaum J.L., Brouwers-Haspels I., Asmawidjaja P.S., Mus A.M., Razawy W., Molendijk M., Clausen B.E., Lubberts E. (2015). Dendritic Cell-Specific Deletion of β-Catenin Results in Fewer Regulatory T-Cells without Exacerbating Autoimmune Collagen-Induced Arthritis. PLoS ONE.

[162] Gattinoni L., Zhong X.S., Palmer D.C., Ji Y., Hinrichs C.S., Yu Z., Wrzesinski C., Boni A., Cassard L., Garvin L.M. (2009). Wnt signaling arrests effector T cell differentiation and generates CD8+ memory stem cells. Nat. Med..

[163] Swafford D., Manicassamy S. (2015). Wnt signaling in dendritic cells: Its role in regulation of immunity and tolerance. Discov. Med..

[164] Xu Y., Banerjee D., Huelsken J., Birchmeier W., Sen J.M. (2003). Deletion of beta-catenin impairs T cell development. Nat. Immunol..

[165] Staal F.J., Luis T.C., Tiemessen M.M. (2008). WNT signalling in the immune system: WNT is spreading its wings. Nat. Rev. Immunol..

[166] Gattinoni L., Ji Y., Restifo N.P. (2010). Wnt/beta-catenin signaling in T-cell immunity and cancer immunotherapy. Clin. Cancer Res..

[167] Van Loosdregt J., Coffer P.J. (2018). The Role of WNT Signaling in Mature T Cells: T Cell Factor Is Coming Home. J. Immunol..

[168] Trujillo J.A., Sweis R.F., Bao R., Luke J.J. (2018). T Cell-Inflamed versus Non-T Cell-Inflamed Tumors: A Conceptual Framework for Cancer Immunotherapy Drug Development and Combination Therapy Selection. Cancer Immunol. Res..

[169] Olson D.J., Luke J.J. (2019). The T-cell-inflamed tumor microenvironment as a paradigm for immunotherapy drug development. Immunotherapy.

[170] Luke J.J., Bao R., Sweis R.F., Spranger S., Gajewski T.F. (2019). WNT/β-catenin Pathway Activation Correlates with Immune Exclusion across Human Cancers. Clin. Cancer Res..

[171] Spranger S., Bao R., Gajewski T.F. (2015). Melanoma-intrinsic β-catenin signalling prevents anti-tumour immunity. Nature.

[172] Spranger S., Gajewski T.F. (2015). A new paradigm for tumor immune escape: β-catenin-driven immune exclusion. J. Immunother. Cancer.

[173] Nsengimana J., Laye J., Filia A., O’Shea S., Muralidhar S., Poźniak J., Droop A., Chan M., Walker C., Parkinson L. (2018). β-Catenin-mediated immune evasion pathway frequently operates in primary cutaneous melanomas. J. Clin. Investig..

[174] Gajos-Michniewicz A., Czyz M. (2016). Modulation of WNT/β-catenin Pathway in Melanoma by Biologically Active Components Derived from Plants. Fitoterapia.

[175] Sferrazza G., Corti M., Brusotti G., Pierimarchi P., Temporini C., Serafino A., Calleri E. (2020). Nature-derived compounds modulating Wnt/β-catenin pathway: A preventive and therapeutic opportunity in neoplastic diseases. Acta Pharm. Sin..

[176] Holtzhausen A., Zhao F., Evans K.S., Tsutsui M., Orabona C., Tyler D.S., Hanks B.A. (2015). Melanoma-Derived Wnt5a Promotes Local Dendritic-Cell Expression of IDO and Immunotolerance: Opportunities for Pharmacologic Enhancement of Immunotherapy. Cancer Immunol. Res..

[177] You L., He B., Xu Z., Uematsu K., Mazieres J., Fujii N., Mikami I., Reguart N., McIntosh J.K., Kashani-Sabet M. (2004). An anti-Wnt-2 monoclonal antibody induces apoptosis in malignant melanoma cells and inhibits tumor growth. Cancer Res..

[178] Fujii N., You L., Xu Z., Uematsu K., Shan J., He B., Mikami I., Edmondson L.R., Neale G., Zheng J. (2007). An antagonist of dishevelled protein-protein interaction suppresses beta-catenin-dependent tumor cell growth. Cancer Res..

[179] Waaler J., Mygland L., Tveita A., Strand M.F., Solberg N.T., Olsen P.A., Aizenshtadt A., Fauskanger M., Lund K., Brinch S.A. (2020). Tankyrase inhibition sensitizes melanoma to PD-1 immune checkpoint blockade in syngeneic mouse models. Commun. Biol..

[180] Molagoda I.M.N., Karunarathne W.A.H.M., Park S.R., Choi Y.H., Park E.K., Jin C.Y., Yu H., Jo W.S., Lee K.T., Kim G.Y. (2020). GSK-3β-Targeting Fisetin Promotes Melanogenesis in B16F10 Melanoma Cells and Zebrafish Larvae Through β-Catenin Activation. J. Mol. Sci..

[181] Syed D.N., Afaq F., Maddodi N., Johnson J.J., Sarfaraz S., Ahmad A., Setaluri V., Mukhtar H. (2011). Inhibition of human melanoma cell growth by the dietary flavonoid fisetin is associated with disruption of Wnt/β-catenin signaling and decreased Mitf levels. J. Investig. Dermatol..

[182] Tarapore R.S., Siddiqui I.A., Saleem M., Adhami V.M., Spiegelman V.S., Mukhtar H. (2010). Specific targeting of Wnt/β-catenin signaling in human melanoma cells by a dietary triterpene lupeol. Carcinogenesis.

[183] Talar B., Gajos-Michniewicz A., Talar M., Chouaib S., Czyz M. (2016). Pentoxifylline Inhibits WNT Signalling in β-Catenin^high^ Patient-Derived Melanoma Cell Populations. PLoS ONE.

[184] Bagheri M., Tabatabae Far M.A., Mirzaei H., Ghasemi F. (2020). Evaluation of antitumor effects of aspirin and LGK974 drugs on cellular signaling pathways, cell cycle and apoptosis in colorectal cancer cell lines compared to oxaliplatin drug. Fundam. Clin. Pharmacol..

[185] Liu J., Pan S., Hsieh M.H., Ng N., Sun F., Wang T., Kasibhatla S., Schuller A.G., Li A.G., Cheng D. (2013). Targeting Wnt-driven cancer through the inhibition of Porcupine by LGK974. Proc. Natl. Acad. Sci. USA.

[186] Madan B., Ke Z., Harmston N., Ho S.Y., Frois A.O., Alam J., Jeyaraj D.A., Pendharkar V., Ghosh K., Virshup I.H. (2016). Wnt addiction of genetically defined cancers reversed by PORCN inhibition. Oncogene.

[187] Li C., Cao J., Zhang N., Tu M., Xu F., Wei S., Chen X., Xu Y. (2018). Identification of RSPO2 Fusion Mutations and Target Therapy Using a Porcupine Inhibitor. Sci. Rep..

[188] El-Khoueiry A.B., Ning Y., Yang D., Cole S., Kahn M., Zoghbi M., Berg J., Fujimori M., Inada T., Kouji H. (2013). A phase I first-in-human study of PRI-724 in patients (pts) with advanced solid tumors. J. Clin. Oncol..

[189] Le P.N., McDermott J.D., Jimeno A. (2015). Targeting the Wnt pathway in human cancers: Therapeutic targeting with a focus on OMP-54F28. Pharmacol. Ther..

[190] Gurney A., Axelrod F., Bond C.J., Cain J., Chartier C., Donigan L., Fischer M., Chaudhari A., Ji M., Kapoun A.M. (2012). Wnt pathway inhibition via the targeting of Frizzled receptors results in decreased growth and tumorigenicity of human tumors. Proc. Natl. Acad. Sci. USA.

[191] Osman J., Bellamkonda K., Liu Q., Andersson T., Sjölander A. (2019). The WNT5A Agonist Foxy5 Reduces the Number of Colonic Cancer Stem Cells in a Xenograft Mouse Model of Human Colonic Cancer. Anticancer Res..

[192] Mehdawi L.M., Prasad C.P., Ehrnström R., Andersson T., Sjölander A. (2016). Non-canonical WNT5A signaling up-regulates the expression of the tumor suppressor 15-PGDH and induces differentiation of colon cancer cells. Mol. Oncol..

[193] Diamond J.R., Eckhardt S.G., Bendell J.C., Munster P., Morris V.K., Kopetz S., Cattaruzza F., Kapoun A.M., Dupont J., Faoro L. A Phase 1a/b study of OMP-131R10, an anti-RSPO3 antibody, in advanced solid tumors and previously treated metastatic colorectal cancer (CRC). Proceedings of the TAT 2016 Conference.

[194] Osada T., Chen M., Yang X.Y., Spasojevic I., Vandeusen J.B., Hsu D., Clary B.M., Clay T.M., Chen W., Morse M.A. (2011). Antihelminth compound niclosamide downregulates Wnt signaling and elicits antitumor responses in tumors with activating APC mutations. Cancer Res..

[195] Mook R.A., Wang J., Ren X.R., Piao H., Lyerly H.K., Chen W. (2019). Identification of novel triazole inhibitors of Wnt/β-catenin signaling based on the Niclosamide chemotype. Bioorg. Med. Chem. Lett..

[196] Leung S.W., Chou C.J., Huang T.C., Yang P.M. (2019). An Integrated Bioinformatics Analysis Repurposes an Antihelminthic Drug Niclosamide for Treating HMGA2-Overexpressing Human Colorectal Cancer. Cancers.

[197] Park S.Y., Kim J.Y., Choi J.H., Kim J.H., Lee C.J., Singh P., Sarkar S., Baek J.H., Nam J.S. (2019). Inhibition of LEF1-Mediated DCLK1 by Niclosamide Attenuates Colorectal Cancer Stemness. Clin. Cancer Res..

[198] Bhattacharyya J., Ren X.R., Mook R.A., Wang J., Spasojevic I., Premont R.T., Li X., Chilkoti A., Chen W. (2017). Niclosamide-conjugated polypeptide nanoparticles inhibit Wnt signaling and colon cancer growth. Nanoscale.

[199] Ahn S.Y., Kim N.H., Lee K., Cha Y.H., Yang J.H., Cha S.Y., Cho E.S., Lee Y., Cha J.S., Cho H.S. (2017). Niclosamide is a potential therapeutic for familial adenomatosis polyposis by disrupting Axin-GSK3 interaction. Oncotarget.

[200] Monin M.B., Krause P., Stelling R., Bocuk D., Niebert S., Klemm F., Pukrop T., Koenig S. (2016). The anthelmintic niclosamide inhibits colorectal cancer cell lines via modulation of the canonical and noncanonical Wnt signaling pathway. J. Surg. Res..

[201] Burock S., Daum S., Keilholz U., Neumann K., Walther W., Stein U. (2018). Phase II trial to investigate the safety and efficacy of orally applied niclosamide in patients with metachronous or sychronous metastases of a colorectal cancer progressing after therapy: The NIKOLO trial. BMC Cancer.

[202] Pintova S., Dharmupari S., Moshier E., Zubizarreta N., Ang C., Holcombe R.F. (2019). Genistein combined with FOLFOX or FOLFOX-Bevacizumab for the treatment of metastatic colorectal cancer: Phase I/II pilot study. Cancer Chemother. Pharmacol..

[203] Pintova S., Planutis K., Planutiene M., Holcombe R.F. (2017). ME-143 Is Superior to Genistein in Suppression of WNT Signaling in Colon Cancer Cells. Anticancer Res..

[204] Nguyen A.V., Martinez M., Stamos M.J., Moyer M.P., Planutis K., Hope C., Holcombe R.F. (2009). Results of a phase I pilot clinical trial examining the effect of plant-derived resveratrol and grape powder on Wnt pathway target gene expression in colonic mucosa and colon cancer. Cancer Manag. Res..

[205] Park C.H., Hahm E.R., Park S., Kim H.K., Yang C.H. (2005). The inhibitory mechanism of curcumin and its derivative against beta-catenin/Tcf signaling. FEBS Lett..

[206] Katoh M., Katoh M. (2017). Molecular genetics and targeted therapy of WNT-related human diseases (Review). Int. J. Mol. Med..

[207] Chen M., Wang J., Lu J., Bond M.C., Ren X.R., Lyerly H.K., Barak L.S., Chen W. (2009). The anti-helminthic niclosamide inhibits Wnt/Frizzled1 signaling. Biochemistry.

[208] Mook R.A., Wang J., Ren X.R., Chen M., Spasojevic I., Barak L.S., Lyerly H.K., Chen W. (2015). Structure-activity studies of Wnt/β-catenin inhibition in the Niclosamide chemotype: Identification of derivatives with improved drug exposure. Bioorg. Med. Chem..

[209] Kagey M.H., He X. (2017). Rationale for targeting the Wnt signalling modulator Dickkopf-1 for oncology. Br. J. Pharmacol..

[210] Wall J.A., Klempner S.J., Arend R.C. (2020). The anti-DKK1 antibody DKN-01 as an immunomodulatory combination partner for the treatment of cancer. Expert Opin. Investig. Drugs.

[211] Fujita S., Mukai T., Mito T., Kodama S., Nagasu A., Kittaka M., Sone T., Ueki Y., Morita Y. (2018). Pharmacological inhibition of tankyrase induces bone loss in mice by increasing osteoclastogenesis. Bone.

[212] Lau T., Chan E., Callow M., Waaler J., Boggs J., Blake R.A., Magnuson S., Sambrone A., Schutten M., Firestein R. (2013). A Novel Tankyrase Small-Molecule Inhibitor Suppresses APC Mutation-Driven Colorectal Tumor Growth. Cancer Res..

[213] Cheng X., Xu X., Chen D., Zhao F., Wang W. (2019). Therapeutic potential of targeting the Wnt/β-catenin signaling pathway in colorectal cancer. Biomed. Pharmacother..

[214] Krishnamurthy N., Kurzrock R. (2018). Targeting the Wnt/beta-catenin Pathway in Cancer: Update on Effectors and Inhibitors. Cancer Treat. Rev..

